# Smoothelin-like 1 deletion enhances myogenic reactivity of mesenteric arteries with alterations in PKC and myosin phosphatase signaling

**DOI:** 10.1038/s41598-018-36564-0

**Published:** 2019-01-24

**Authors:** Sara R. Turner, Mona Chappellaz, Brittany Popowich, Anne A. Wooldridge, Timothy A. J. Haystead, William C. Cole, Justin A. MacDonald

**Affiliations:** 1 Department of Biochemistry & Molecular Biology, Cumming School of Medicine, University of Calgary, Calgary, AB T2N 4Z6 Canada; 2 Department of Pharmacology & Cancer Biology, Duke University School of Medicine, Durham, NC 27710 USA; 3 Department of Physiology & Pharmacology, Cumming School of Medicine, University of Calgary, Calgary, AB T2N 4N1 Canada; 4 Present Address: Department of Clinical Sciences, College of Veterinary Medicine, Auburn University, Auburn, AL 36849 USA

## Abstract

The role of the smoothelin-like 1 (SMTNL1) protein in mediating vascular smooth muscle contractile responses to intraluminal pressure was examined in resistance vessels. Mesenteric arterioles from wild type (WT) and SMTNL1 global knock-out (KO) mice were examined with pressure myography. SMTNL1 deletion was associated with enhanced myogenic tone in vessels isolated from male, but not female, mice. Intraluminal pressures greater than 40 mmHg generated statistically significant differences in myogenic reactivity between WT and KO vessels. No overt morphological differences were recorded for vessels dissected from KO animals, but SMTNL1 deletion was associated with loss of myosin phosphatase-targeting protein MYPT1 and increase in the myosin phosphatase inhibitor protein CPI-17. Additionally, we observed altered contractile responses of isolated arteries from SMTNL1 KO mice to phenylephrine, KCl-dependent membrane depolarization and phorbol 12,13-dibutyrate (PDBu). Using pharmacological approaches, myogenic responses of both WT and KO vessels were equally affected by Rho-associated kinase (ROCK) inhibition; however, augmented protein kinase C (PKC) signaling was found to contribute to the increased myogenic reactivity of SMTNL1 KO vessels across the 60–120 mmHg pressure range. Based on these findings, we conclude that deletion of SMTNL1 contributes to enhancement of pressure-induced contractility of mesenteric resistance vessels by influencing the activity of myosin phosphatase.

## Introduction

As a member of the smoothelin family of muscle proteins, the 459-amino acid smoothelin-like 1 protein (SMTNL1; initially termed CHASM, calponin homology-associated smooth muscle^1,2^) is characterized by the presence of a C-terminal calponin homology (CH)-domain, also found in the other smoothelins^3,4^. The smoothelins are specifically expressed in, and frequently used as markers of, differentiated contractile smooth muscle cells^3^; however, findings discriminate SMTNL1 functionally from the other smoothelin proteins^4^. SMTNL1 appears to provide a potential mechanism for receptor-mediated signaling that promotes changes in vascular smooth muscle contractility through regulation of myosin light chain phosphatase (MLCP) activity^2,5^. *In vitro* biochemical studies showed that SMTNL1 (in its dephosphorylated state) could directly inhibit MCLP activity. It is still unclear how exactly SMTNL1 acts to inhibit MLCP; however, the effect was relieved when SMTNL1 was phosphorylated at Ser301. In addition, newer data suggest that SMTNL1 may drive long-term regulation of contractile capacity through transcriptional or translational effects on the expression levels of MYPT1 (the myosin phosphatase-targeting subunit of MLCP) as well as other targets involved in muscle plasticity^6–8^. Finally, studies reveal a regulatory capacity for SMTNL1 in promoting the sex-dependent physiological adaptations to different environmental influences. This may be reflective of transcriptional distinctions for SMTNL1 between sexes: in adult female mice, SMTNL1 protein content is ~50–70% of that for males^8^. In this regard, SMTNL1 content increases throughout sexual development, and the difference in the expression levels observed between the sexes persists regardless of age.

The impact of SMTNL1 on vascular smooth muscle was previously studied using isolated aortic smooth muscle rings from wild type (WT) and knock-out (KO) mice. In the absence of SMTNL1, vascular smooth muscle was less responsive to contractile agonists (e.g., phenylephrine, PE) and was more responsive to relaxant agonists (i.e., acetylcholine, ACh & nitric oxide, NO)^5^. SMTNL1 also appeared to play a role in the adaptive response of vascular smooth muscle to exercise. In this context, exercise itself reduced SMTNL1 expression, and vascular contractility in a SMTNL1 KO mouse was reflective of a cardiovascular phenotype achieved following endurance-exercise training. Notably, male KO mice demonstrated more willingness to complete treadmill running, had an increased time to fatigue, and also required fewer motivational stimuli throughout a 5-week exercise protocol. Sedentary male KO mice displayed more robust exercise performance of skeletal muscle and improved cardiovascular responses when compared to WT animals^5^. Consistent with sex dimorphism in SMTNL1-dependent adaptations to exercise, the differences in cardiovascular performance generated by SMTNL1 knock-out in female mice were less remarkable.

The small resistance arteries (i.e., <250 μm i.d.) regulate total peripheral resistance through intrinsic tone development that originates within the vascular smooth muscle as a response to transmural pressure and circumferential wall-stress (i.e., the myogenic response)^9^. Most studies of SMTNL1 in the vasculature, to date, have been conducted with aortic rings^5,8^, although pilot studies implicate a role for SMTNL1 in the contractility of the cerebral microvasculature^10^. While the aorta provides ample tissue for biochemical analysis, generates strong contractions, and is a relatively easy tissue to dissect and mount in tissue baths, it is ultimately a conduit vessel, which contributes only a small amount to peripheral resistance and establishment of blood pressure^11^. To better understand the contribution of SMTNL1 to vascular autoregulation of blood flow, a resistance vessel bed that exhibits myogenic reactivity was selected. The mesenteric arteries are capable of dramatically regulating flow in response to digestion or fight-or-flight activity and demonstrate the full potential of myogenic tone and blood flow regulation^12^. The availability of third- and fourth-order mesenteric vessels within the splanchnic circulation provides sufficient tissue for protein extraction and biochemical assessments. Furthermore, the mesenteric arteries at rest receive more than 10% of the total cardiac output, and are therefore a significant contributor to basal peripheral resistance^13^. As such, the primary objective of this study was to complete an *ex vivo* investigation of the role of SMTNL1 in vascular contractility and the myogenic response of mesenteric arterioles isolated from SMTNL1 WT and KO mice, and in some cases, male and female animals.

## Materials and Methods

### Chemicals

All chemicals were of reagent grade or higher, and unless otherwise stated were purchased from Millipore-Sigma (Etobicoke, ON) or VWR (Mississauga, ON). Antibodies were obtained from Santa Cruz Biotechnology (Dallas, TX: anti-LC20, sc-15370; anti-MYPT1, H-130), Abcam (Toronto, ON: anti-smooth muscle α-actin, ab7817; anti-MLCK, ab76092), Millipore-Sigma (anti-CPI-17, 07–344), Chemicon (Temecula, CA: horseradish peroxidase (HRP)-coupled donkey anti-rabbit IgG). The ROCK inhibitor, GSK269962A (PubChem CID: 16095342), was acquired from Alexis Biochemicals (San Diego, CA). Bradykinin, phenylephrine, phorbol 12,13-dibutyrate (PDBu), and the PKC inhibitor GF109203x (PubChem CID: 2396) were purchased from Millipore-Sigma.

### Animals

Wild-type (WT) and *Smtnl1* knockout mice (KO, global: previously described)^5^ on a 129S6/SvEvTac background were bred and housed at the University of Calgary. Some experiments used C57Bl/6 mice that were purchased from Charles River Laboratory (Montreal, QC). Adult mice used in the studies were sacrificed at 10–12 weeks of age. All protocols for animal experimentation were approved by the Animal Care and Use Committee at the University of Calgary and conform to the guidelines set by the Canadian Council of Animal Care.

### Tissue collection

Animals were euthanized by isoflurane inhalation to the point of being fully anesthetized, followed by cervical dislocation. Immediately following euthanasia, the gut from the duodenum to the cecum, removed intact to preserve the mesenteric arterial network required for study, was removed by gross dissection and placed into room temperature normal Krebs’ buffer (NB) containing: 120 mM NaCl, 25 mM NaHCO_3_, 4.8 mM KCl, 1.2 mM NaH_2_PO_4_, 1.2 mM MgSO_4_, 11 mM glucose and 1.8 mM CaCl_2_ (pH 7.4). Other tissues collected to study vascular beds included: the brain (removed intact to preserve the middle and posterior cerebral arteries, MCA and PCA respectively), the thoracic aorta, the femoral artery, and the tail (marked to identify the location of the caudal artery).

### Vessel isolation and mounting for pressure myography

Third-order mesenteric arteries (MA) were collected and used for pressure myography. The MAs were identified by pinning out the ileum from the cecum in a circle; in this arrangement, the first-order mesenteric arteries branch from the superior mesenteric artery in the center with the third-order vessels being the third branch moving proximal to the ileum. Vessels were cleaned of the surrounding tissue, cut into 3–5 mm segments, mounted on a glass cannula in an arteriograph chamber (Living Systems), and tied in place with two pieces of silk suture. To denude the vessel of endothelial cells, each vessel was passed fully on the glass cannula prior to tying in place, and then a stream of air bubbles was passed through the lumen of the vessel. The vessel chamber was then connected to the pressure myograph (Living Systems) for measurement of vessel diameter with an automated edge detection system (IonOptix). Vessels were briefly pressurized to 80 mmHg (less than 1 minute), and leaky vessels were either retied or discarded. Pressure was then set to 10 mmHg and vessels were warmed to 37 °C by perfusion with warm, aerated (with 95% air/5% CO_2_) NB for a 15–20-minute equilibration period. Confirmation of endothelial removal was evaluated via loss of vasodilatory response to acetylcholine (ACh, 1 µM) in vessels with myogenic constrictions at 80 mmHg.

### Pressure-induced constriction protocol

To evaluate the myogenic response, mounted vessels were first tested for the presence of myogenic contractions, as the myogenic response may be lost due to mishandling during dissection and mounting. Vessels were pressurized to 80 mmHg, and the pressure was dropped back to 10 mmHg following development of myogenic constrictions and stabilization of vessel diameters. The single step to 80 mmHg was repeated up to two additional times to confirm the consistency of the myogenic response. The vessel was then subjected to pressure steps in NB, from 10 to 120 mmHg; the individual pressure steps lasted for ≥5 minutes to allow for the development of stable myogenic tone. Pressure was then returned to 10 mmHg. In cases where potential inhibitors of smooth muscle contraction were tested for effects on the myogenic response, compounds were added to the bath superfusate; the vessel was then incubated at 10 mmHg for 30 minutes prior to repeating the 10–120 mmHg pressure steps. Pressure was then returned to 10 mmHg and the NB, with or without inhibitor, was removed and replaced with Ca^2+^-free NB (same constitution as NB but with no CaCl_2_ and 2 mM EGTA added). Data were collected as average vessel diameter during stable development at each pressure step and were expressed as a percentage of the maximal passive vessel diameter in Ca^2+^-free NB at 120 mmHg to standardize for variations in the size of vessels among animals.

### Calculation of structural and mechanical properties of small mesenteric arteries

Vessel wall thickness (VWT), cross-sectional area (CSA), and vessel wall/lumen (VW/L) ratio were calculated as follows: VWT = (d_e_ − d_i_)/2; CSA = (π/4) × [(d_e_)2 − (d_i_)2]; and VW/L = (d_e_ −  d_i_)/2d_e_, where d_e_ and d_i_ are the external and internal diameters observed for a given intravascular pressure under passive conditions (Ca^2+^-free NB), respectively. Mechanical parameters were calculated as previously described^14,15^. Circumferential wall strain (ɛ) was calculated as (d_i_ − d_i/0_)/d_i/0_, where d_i_ is the observed internal lumen diameter for a given intravascular pressure and d_i/0_ is the internal diameter at very low pressure value (i.e., 3 mmHg), both measured under passive conditions. Circumferential wall stress (σ) was determined as (Px × d_i_)/2VWT, where Px is the intraluminal pressure (1 mmHg = 0.133 kPa) and VWT is wall thickness at each intraluminal pressure in Ca^2+^-free medium. Incremental distensibility was evaluated in Ca^2+^-free NB by lowering the intramural pressure to 3 mm Hg so that the vessel was inflated but unstressed. Intramural pressure was then increased in a stepwise fashion up to 120 mmHg. Lumen diameter was allowed to stabilize for 1 min after each increase in pressure, and distensibility was calculated at each pressure (Px) as a percentage of the lumen diameter for each 1 mmHg change in the intralumenal pressure [Δd_i_/(d_i_ at 3 mm Hg × ΔP) × 100].

### Sample collection and protein extraction

Proteins were extracted by heating flash-frozen vessels in SDS-sample buffer (containing 4% (w/v) SDS, 100 mM DTT, 10% (v/v) glycerol, and 60 mM Tris-HCl pH 6.8) at 95 °C for 10 minutes. For vessels smaller than 500 µm in diameter, tissues were placed into 1.5 ml microtubes with 30–50 µl of SDS-sample buffer. Samples were briefly centrifuged to ensure tissues were fully submerged in buffer and were then vortexed overnight at 4 °C. Larger tissue samples (e.g., aorta, femoral and caudal arteries) were homogenized by hand in a ground-glass homogenizer in 100 µl of SDS-sample buffer. The homogenate volume was increased to 500 µl with addition of SDS-sample buffer, and then the samples were vortexed overnight at 4 °C. All tissue extracts were stored at −20 °C before use.

### Western blotting

Proteins were separated by SDS-PAGE and then were transferred to nitrocellulose membranes (0.2 µm pore size, Bio-Rad) at 25 V (constant voltage) for 16 hours overnight at 4 °C in standard Tris-glycine-methanol buffer. For CPI-17, a 10 mM N-cyclohexyl-3-aminopropanesulfonic acid (CAPS, pH 11) with 10% (v/v) methanol buffer was used. Following transfer, proteins were cross-linked to the membrane with 0.25% (v/v) glutaraldehyde in PBS for 30 minutes at room temperature. MYPT1, LC20, and MLCK levels were determined by conventional western blotting (primary antibody: 1:1,000 dilution). CPI-17 levels were assessed with a three-step western blotting protocol. A biotin-conjugated secondary antibody (Chemicon; 1:10,000 dilution in TBST) was incubated with the membrane for 1 hour at room temperature, after which, the membrane was washed repeatedly. A third step to further amplify signal was conducted with incubation of membrane in HRP-conjugated streptavidin (Pierce; 1:20,000 dilution). Signals were developed with either ECL (GE Healthcare) or SuperSignal West Femto (Pierce) according to the manufacturer’s directions. The emitted chemiluminescence was detected with a LAS4000 imaging analyzer (GE Healthcare) and obtained images were analyzed by densitometry with ImageQuant software (GE Healthcare). Protein expression was normalized to α-smooth muscle actin content in each sample.

### Statistical analysis

All data are presented as mean ± standard error of the mean (SEM). The n values for isolated vessels represent the number of vessels examined, with each vessel originating from a distinct animal. Statistical significance was determined with GraphPad Prism v7.0 software using the Student’s *t*-test (two-tailed), or two-way ANOVA with Tukey’s or Sidak’s multiple comparison *post hoc* tests, as appropriate, with P < 0.05 used as the cut off.

## Results

### Effect of SMTNL1 KO on pressure-evoked myogenic constrictions of mesenteric arteries

**Figure 1:**
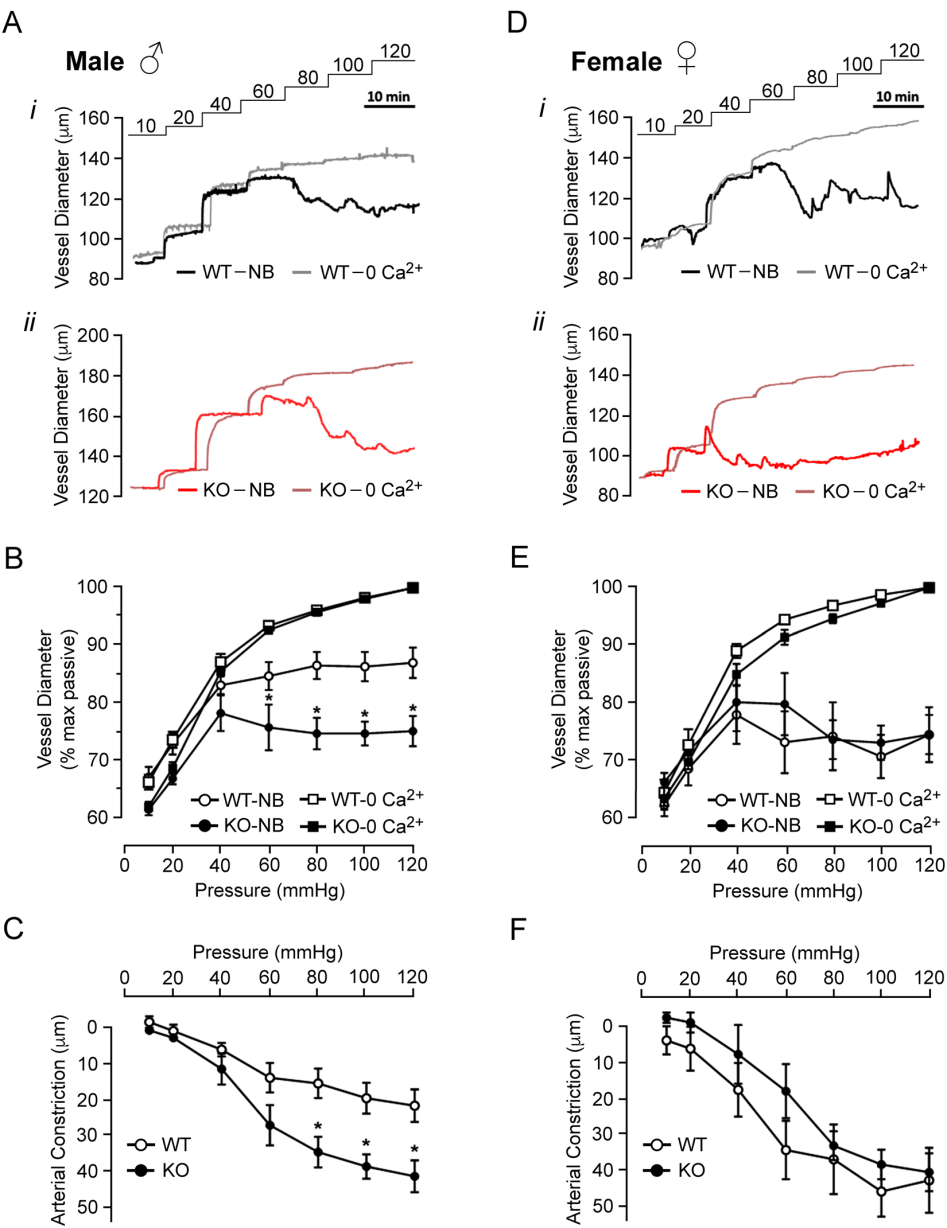
SMTNL1 deletion enhances myogenic responsiveness of male mesenteric arteries. Representative pressure myography recordings are provided for outer vessel diameters of third-order mouse mesenteric arteries subjected to sequential pressure steps (10–120 mmHg) in normal Krebs’ buffer (NB) and Ca^2+^-free buffer (0 Ca^2+^). Vessels were dissected from 10-week-old male (**A**) and female (**D**) mice: WT *SMTNL1*^+/+^(*panel i*) or KO *SMTNL1*^−/−^ (*panel ii*). Cumulative data for the myogenic responses of vessels are expressed as % maximum passive diameter (male, **B**; female, **E**) or the magnitude of vessel constriction (male, **C**; female, **F**). The value for arterial constriction is the difference between WT or KO diameter and the corresponding passive diameter for a given luminal pressure. Results were analyzed by two-way ANOVA and Tukey’s multiple comparison’s test; n = 14 for WT males, n = 10 for KO males, n = 6 for WT females, and n = 4 for KO females. *Indicates statistically significant differences between WT and KO values (p < 0.05).

Standard pressure myography was used to assess the myogenic response of endothelium-denuded mesenteric resistance vessels evoked by step-wise increases in intraluminal pressure from 10 to 120 mmHg. Arteries of 10–12 week WT *SMTNL1*^+/+^ male mice exhibited modest myogenic behavior (i.e., vessel diameter increased proportionally with pressure until ~60 mmHg), and active myogenic constriction was detected above ~80 mmHg with maintenance of a constant pressure–diameter relationship between 80 and 120 mmHg (Fig. 1A, panel i). Given lower than expected myogenic reactivity was recorded when compared to previous publications, the pressure-evoked constrictions of endothelium-denuded mesenteric vessels from 10–12 week C57Bl/6 mice were also assessed (Supplemental Fig. 1). Third-order mesenteric vessels from male C57Bl/6 mice were larger than those isolated from WT *SMTNL1*^+/+^ mice at 191.4 ± 8.1 μm versus 163.8 ± 6.1 μm (p = 0.042, Student’s *t*-test). In this case, the myogenic behaviour was consistent with literature reports for mesenteric arteries obtained from the C57Bl/6 strain of mice^16–20^. Vessels from male C57Bl/6 mice were considerably more myogenic than corresponding vessels from the WT male *SMTNL1*^+/+^ mice on the 129S6/Sv background; the cumulative data reflect increased magnitudes of percentage change relative to the maximum passive diameter in Ca^2+^-free buffer, arterial constriction, and percent myogenic tone development (Supplemental Fig. 1). The molecular origins of the difference in myogenicity between the two mouse strains, and the SMTNL1 expression status in mesenteric arteries of the C57Bl/6 mouse were not explored.

In contrast to WT mice, mesenteric arteries from 10–12 week, male KO *SMTNL1*^−/−^ mice exhibited a significant increase in basal myogenic tone commencing at 60 mmHg and a more pronounced negative slope in the pressure–diameter relationship between 40 and 80 mmHg (Fig. 1A, panel ii). Vessels had similar maximum passive diameters, with no statistically significant difference between WT and KO groups (*p* > 0.05, one-way ANOVA; Fig. 1B). WT vessels had pressure-induced constrictions at 60 mmHg that reached 84.8 ± 2.4% of the maximum passive diameter. A maximal reduction in arterial diameter of 21.8 ± 4.5 μm was developed at 120 mmHg (Fig. 1C). Vessels from KO males had a more robust myogenic response, initiating constrictions at 40 mmHg and reaching 75.9 ± 3.9% of their maximum vessel diameters at 60 mmHg (Fig. 1B). A maximal reduction in arterial diameter was also developed at 120 mmHg, in this case providing a myogenic constriction of 41.5 ± 4.5 μm (Fig. 1C). All pressures greater than 60 mmHg generated statistically significant differences in myogenic reactivity between WT and KO vessels (two-way ANOVA, p < 0.05).

Third-order mesenteric arteries from 10–12 week, female KO animals responded in a similar manner to those collected from the female WT *SMTNL1*^+/+^ (Fig. 1D, panels i and ii); however, vessels from both female WT and KO mice displayed enhanced myogenic reactivity when compared to the vessels obtained from male WT mice. In this case, myogenic responses were initiated in female vessels at 40 mmHg with a negative slope in the pressure–diameter relationship between 40 and 100 mmHg (Fig. 1E). Arterial diameters at 60 mmHg reached 73.2 ± 5.4% and 79.9 ± 5.4% of the maximum passive vessel diameters measured for WT and KO females, respectively. No significant difference in the active myogenic constriction observed at 120 mmHg was observed between female WT and KO vessels, 40.7 ± 9.0 μm and 38.4 ± 5.2 respectively (Fig. 1F).

To evaluate whether deletion of SMTNL1 resulted in any alteration in the mesenteric vascular tree anatomy, intact mesenteric trees were dissected from the superior mesenteric artery to the ileum and examined. The arterial branching network of first- to fourth-order mesenteric vessels was visually inspected. No overt morphological differences were observed between WT and KO animals although branching patterns did vary somewhat between individual animals (Supplemental Fig. S2). Third-order mesenteric arteries were prepared for histological examination, and H&E-stained vessels showed no overt morphological differences (Supplemental Fig. S3). Immunostaining of α-smooth muscle actin indicated that both WT and KO vessels were comprised of 1–2 layers of smooth muscle cells as well as an endothelial cell layer (determined by the α-actin negative, DAPI counterstained luminal cells). Considering the effects of SMTNL1 on myogenic contractility, parameters of vessel wall dynamics were evaluated in tissues isolated from male animals and pressurized over a range of 10–120 mmHg in Ca^2+^-free buffer. There were no statistical differences between WT and KO vessels for inner (luminal) diameter, vessel wall thickness, vessel distensibility, or the circumferential stress/strain relationship, indicating that vascular eutrophic or hypertrophic remodeling processes may be excluded as a factor in the altered myogenic responses observed for male *SMTNL1*^−/−^ mesenteric vessels (Supplemental Fig. S3).

**Figure 2:**
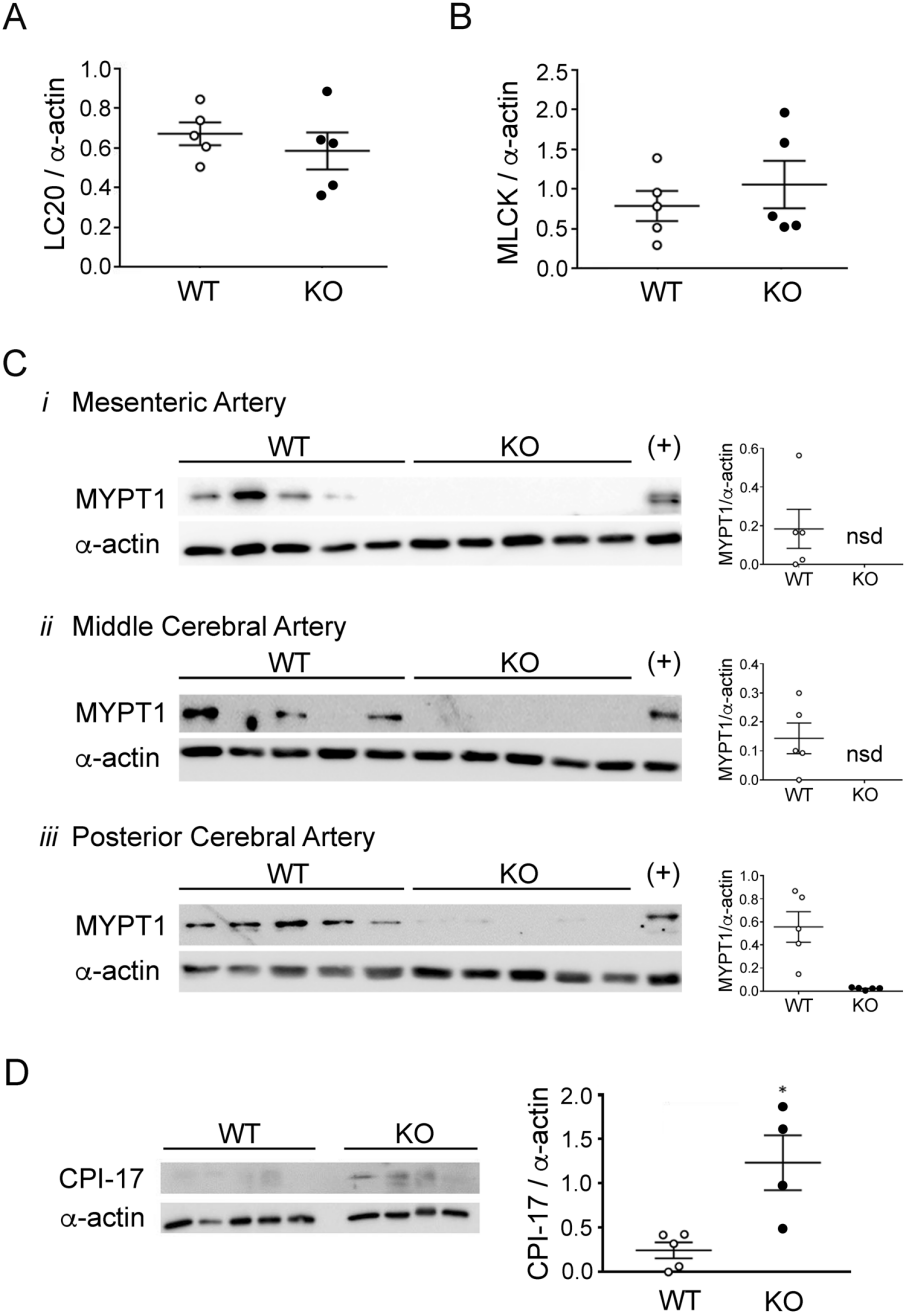
Effect of SMTNL1 deletion on LC20, MLCK, MYPT1 and CPI-17 proteins in the mouse mesenteric vasculature. The levels of myosin regulatory light chain (LC20, **A**) and myosin light chain kinase (MLCK, **B**) were examined by western blotting of mesenteric arteries. Myosin phosphatase-targeting subunit (MYPT1, **C**) was examined in third-order mesenteric arteries as well as middle and posterior cerebral arteries (panels i–iii, respectively). The C-kinase potentiated inhibitor of myosin phosphatase (CPI-17, **D**) was examined by three-step western blotting of mesenteric arteries. All sample lanes were contiguous and originate from the same gel/blot. In the case of MYPT1, the membrane was cut into a “top” and “bottom” so that MYPT1 and α-actin could be independently probed with primary antibodies. Blots are cropped in the vertical axis, so full-size blots are included in the Supplementary Information file. In all cases, arterial tissues were isolated from 10-week old, male WT *SMTNL1*^+/+^ or KO *SMTNL1*^−/−^ mice. *Statistically significant differences between WT and KO mice (p < 0.05, Student’s *t*-test).

Given previous reports for SMTNL1-dependent regulation of gene expression in smooth muscle tissues^6,8^, we examined the levels of important regulatory proteins in a variety of arteries from adult male WT and KO animals. Levels of LC20 and MLCK proteins were not affected by SMTNL1 deletion in any of the vessels examined, including third-order mesenteric vessels (Fig. 2A,B, respectively) as well as aorta, femoral and caudal arteries (Supplemental Fig. S4). The expression of the myosin phosphatase regulatory subunit (MYPT1) was previously linked to SMTNL1 levels^8^. Interestingly, MYPT1 protein was below detection levels in resistance arteries of KO *SMTNL1*^−/−^ mice (n = 5 separate animals when a three-step western blot method was employed), including the mesenteric arteries (Fig. 2C, panel i) as well as middle and posterior cerebral arteries (Fig. 2C, panels ii and iii, respectively). In contrast, MYPT1 protein was detected in the majority of tissue obtained from WT resistance arteries. The KO of SMTNL1 in larger, conduit arteries resulted in more variable MYPT1 levels with no significant effect of SMTNL1 KO on MYPT1 protein (Supplemental Fig. S5). The amount of C-kinase potentiated myosin phosphatase inhibitor of 17-kDa (CPI-17) protein was also impacted by SMTNL1 presence as well as vessel type. In mesenteric arteries (Fig. 2D), CPI-17 levels were elevated approximately 5-fold coincident with SMTNL1 deletion. In the larger conduit vessels (i.e., aorta, femoral and caudal arteries), robust levels of CPI-17 protein were detected with minor enhancement in aorta with SMTNL1 KO, and no significant effect of SMTNL1 deletion impacting upon CPI-17 levels in the other vessels (Supplemental Fig. S6).

**Figure 3:**
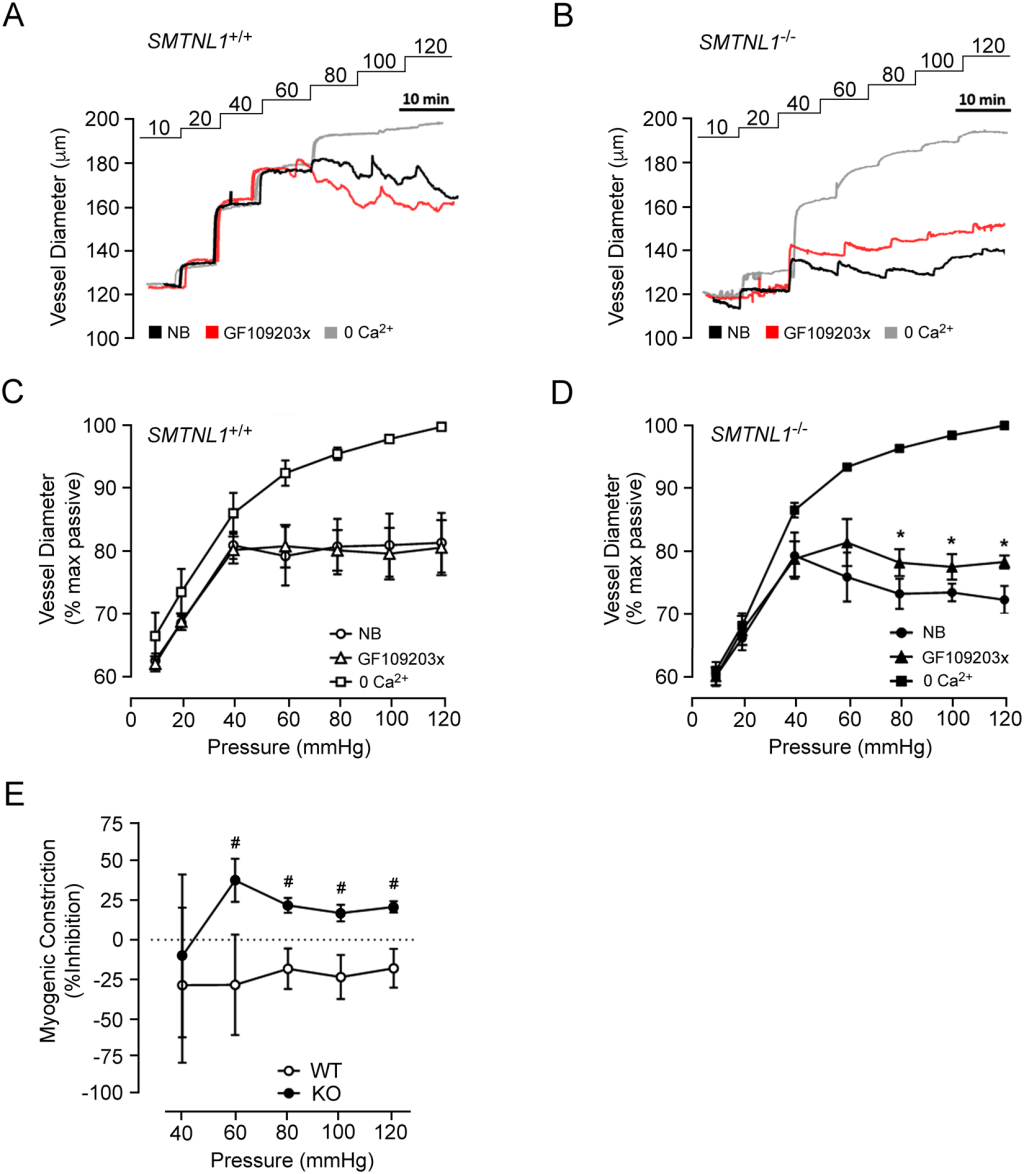
PKC inhibition selectively reduces myogenic responses of KO *SMTNL1*^−/−^ mesenteric vessels. Representative recordings of third-order mesenteric arteries from 10-week old, male WT *SMTNL1*^+/+^ (**A**) and KO *SMTNL1*^−/−^ (**B**) mice subjected to three sequential series of pressure steps (10–120 mmHg) in normal Krebs buffer (NB), with PKC inhibitor (GF109203x, 3 μM) in NB, and in a Ca^2+^-free Krebs buffer (0 Ca^2+^). Cumulative data expressed as the changes in % maximum passive diameter are provided for vessels isolated from WT (**C**) and KO (**D**) animals, showing mean ± SEM (n = 3 separate animals). The effect of GF109203x treatment as the % inhibition of myogenic tone is provided in (**E**). The values for myogenic constrictions were calculated as the difference between WT or KO diameter (in the absence/presence of GF109203x) and the corresponding passive diameter for a given luminal pressure. Results were analyzed by two-way ANOVA and Sidak’s multiple comparisons test, *statistically significant differences between NB and GF109203x treatment (p < 0.05). ^#^Statistically significant differences for PKC-dependent inhibition of myogenic tone between WT and KO (p < 0.05).

**Figure 4:**
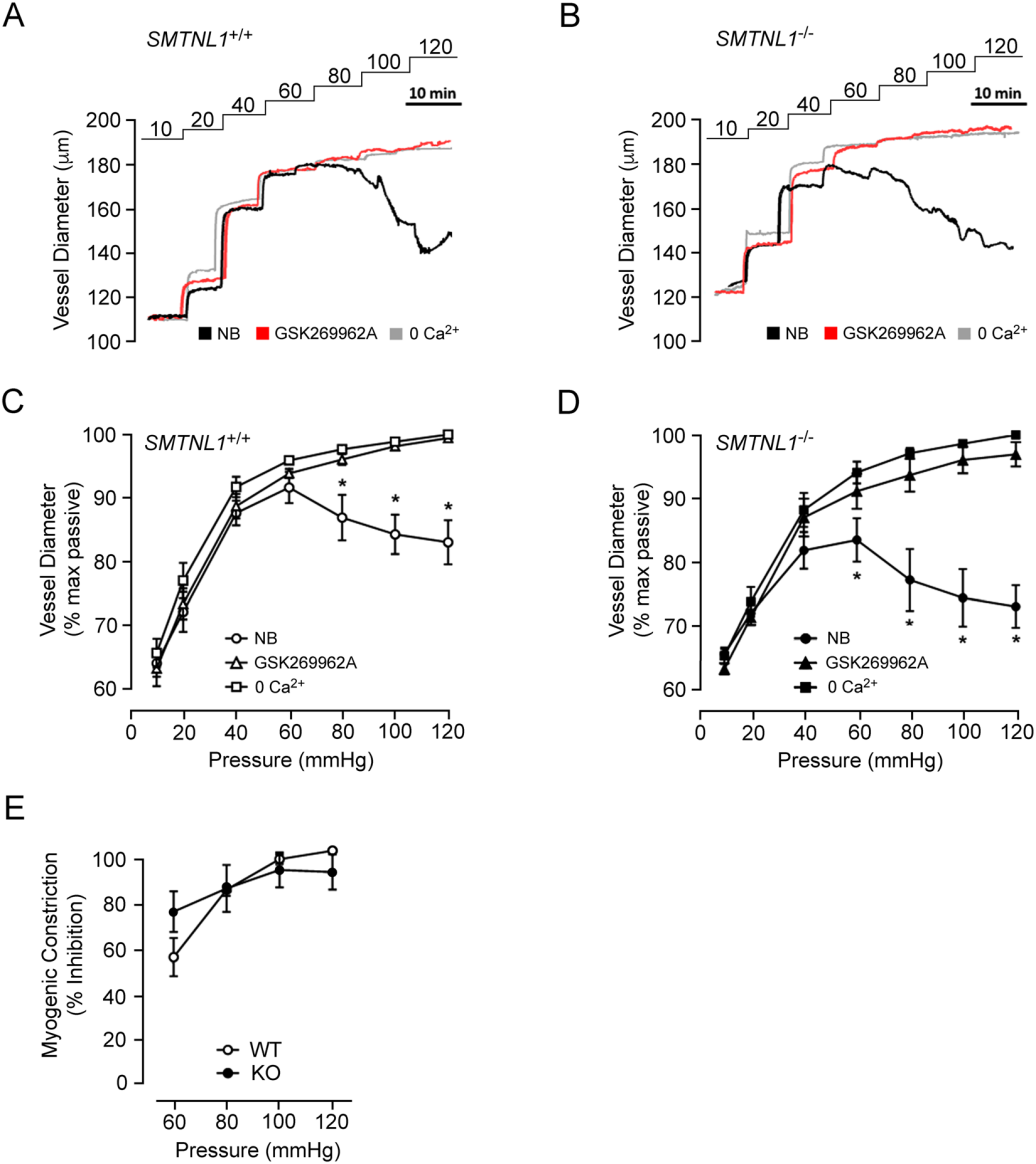
ROCK inhibition abolishes myogenic responses of both WT *SMTNL1*^+/+^ and KO *SMTNL1*^−/−^ mesenteric vessels. Representative recordings of third-order mesenteric arteries, from 10-week old, male WT *SMTNL1*^+/+^ (**A**) and KO *SMTNL1*^−/−^ (**B**) mice, subjected to three sequential series of pressure steps (10–120 mmHg) in normal Krebs buffer (NB), with ROCK inhibitor (GSK269962A, 3 μM) in NB, and in a Ca^2+^-free Krebs buffer (0 Ca^2+^). Cumulative data expressed as the changes in % maximum passive diameter are provided for vessels isolated from WT (**C**) and KO (**D**) animals, showing mean ± SEM (n = 3 separate animals). The effect of GSK269962A treatment as the % inhibition of myogenic tone is provided in (**E**). The values for myogenic constrictions were calculated as the difference between WT or KO diameter (in the absence/presence of GSK269962A) and the corresponding passive diameter for a given luminal pressure. Results were analyzed by two-way ANOVA and Sidak’s multiple comparisons test, *statistically significant differences between NB and ROCK inhibition conditions (p < 0.05). No statistically significant difference was identified for ROCK-dependent inhibition of myogenic tone between WT and KO (p > 0.05).

The increase in CPI-17 protein expression observed in mesenteric arteries of KO *SMTNL1*^−/−^ mice warranted further investigation of contractile signaling pathways acting on the myogenic response of the vessel. While understanding of the regulatory control of CPI-17 by protein kinase pathways is not yet complete, both Ca^2+^- and phospholipid-dependent protein kinase (PKC) and Rho-associated coiled-coil containing protein kinase (ROCK) are suggested to be the major regulators of CPI-17 phosphorylation^21–23^. Moreover, CPI-17 expression is positively correlated with the extent of phorbol ester-induced Ca^2+^ sensitization and contractile force development of vascular smooth muscle^22^. Therefore, we examined the effect of a potent PKC-inhibitor, the GF109203x compound, on the myogenic response of WT and KO mesenteric arteries from male mice. In WT vessels, there was no observable impact of PKC inhibition on myogenic tone (Fig. 3A,C); however, there was a significant attenuation of myogenic reactivity in vessels isolated from the KO *SMTNL1*^−/−^ animals (Fig. 3B,D). In this case, no effect on myogenic responses at low pressures (10 to 40 mmHg) was observed while suppression of myogenic responses was detected at intraluminal pressures greater than ~80 mmHg (Fig. 3B,D). Treatment of KO vessels with GF109203x provided ~50% inhibition of myogenic tone from 60 to 120 mmHg (Fig. 3E). In contrast, ROCK inhibition with GSK269962A was associated with abolishment of myogenic constrictions for both WT and KO vessels at pressures above 60 mmHg (Fig. 4). In this case, the results indicate that when myogenic tone was present, it could be effectively abrogated with addition of ROCK inhibitor, and this effect was independent of SMTNL1 deletion.

**Figure 5:**
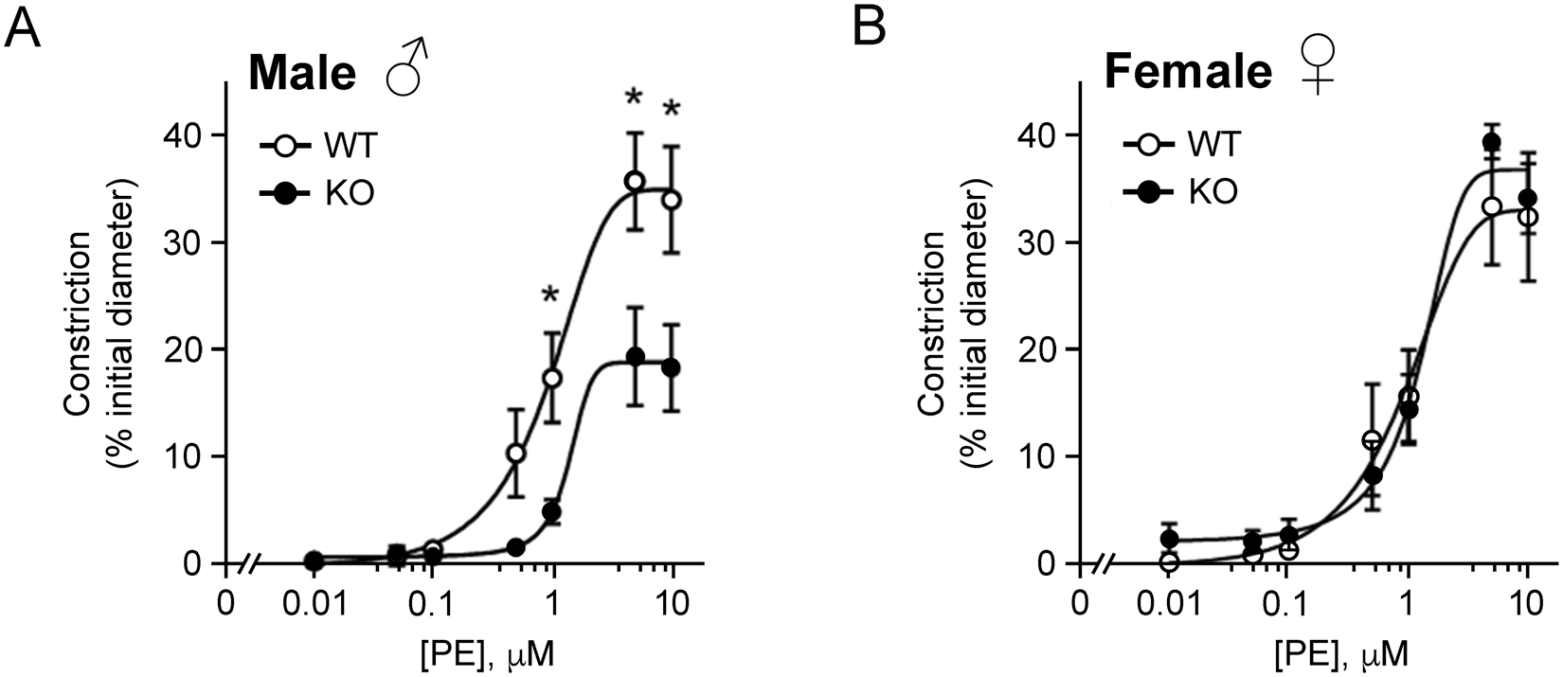
Impact of sex and SMTNL1 knock-out on vasoconstrictive responses of mesenteric arteries to phenylephrine. Third-order mesenteric arteries from 10-week old, male (**A**) or female (**B**) WT *SMTNL1*^+/+^ and KO *SMTNL1*^−/−^ mice were pressurized to 20 mmHg and treated with increasing concentrations of phenylephrine (PE, 10 nM − 10 μM) in the bath superfusate. Cumulative data (% constriction relative to the initial vessel diameter) were fit with sigmoidal concentration-response curves. Results were analyzed by two-way ANOVA and Sidak’s multiple comparisons test, *statistically significant differences between individual WT and KO data points (p < 0.05, n = 5 separate animals).

**Figure 6:**
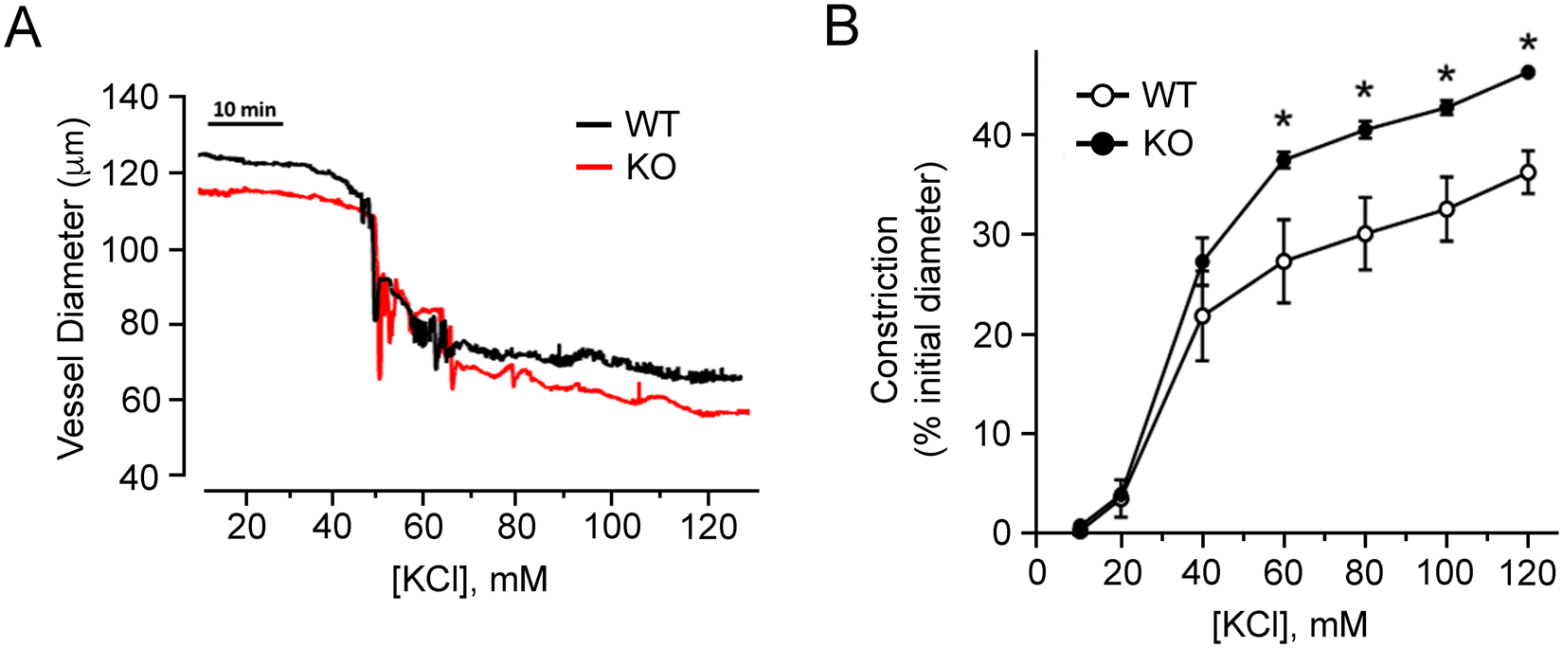
Deletion of SMTNL1 results in mesenteric artery hypercontractility in response to KCl-induced depolarization. Representative recordings of third-order mesenteric arteries from 10-week old, male WT *SMTNL1*^+/+^ and KO *SMTNL1*^−/−^ mice. In (**A**), arteries were pressurized *ex vivo* to 20 mmHg and treated with increasing concentrations of KCl (10–120 mM). Cumulative data (% constriction relative to the initial vessel diameter, **B**) were fit with sigmoidal concentration-response curves. Results were analyzed by two-way ANOVA and Sidak’s multiple comparisons test, *statistically significant differences between individual WT and KO data points (p < 0.05, n = 4 separate animals).

**Figure 7:**
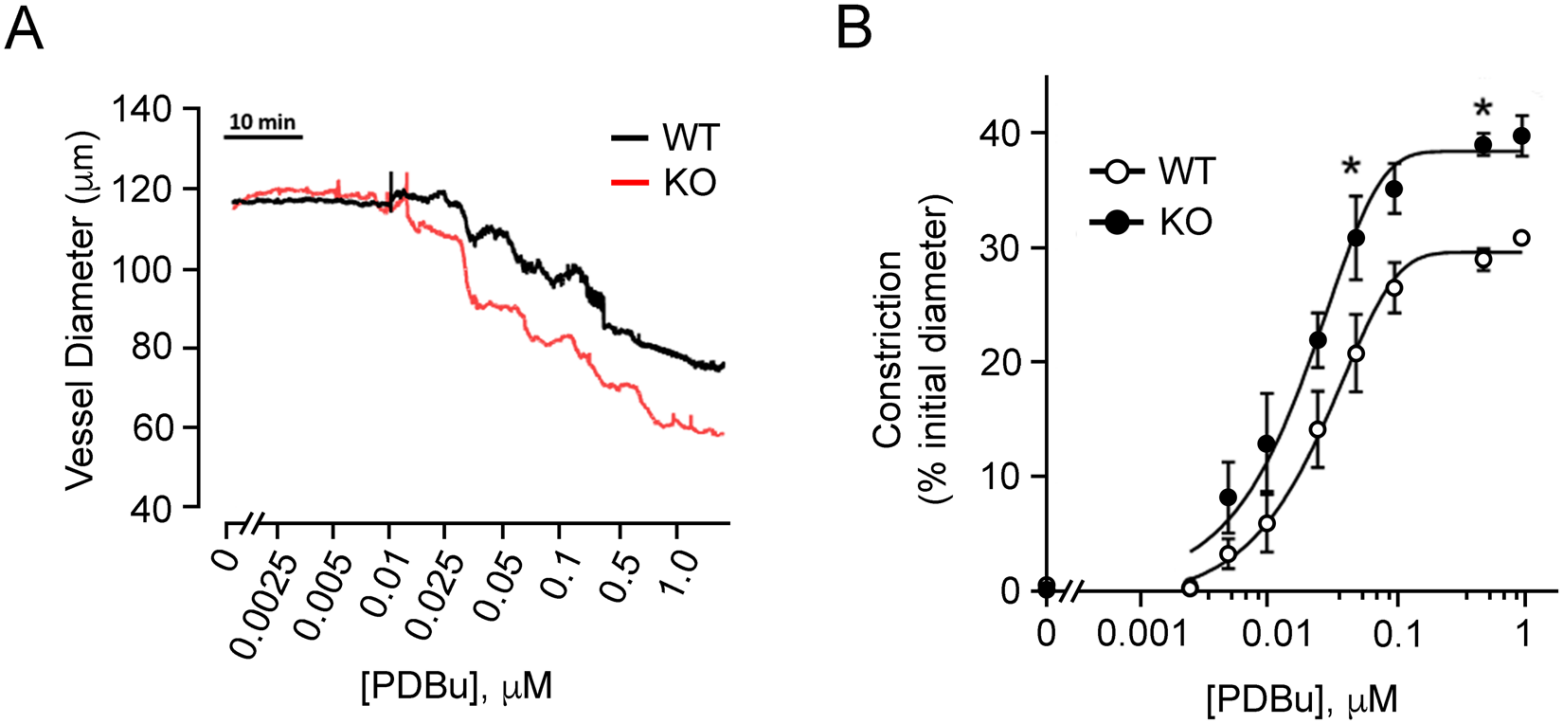
SMTNL1 deletion results in hypercontractility of mesenteric arteries in response to treatment with phorbol ester. Representative recordings of third-order mesenteric arteries from 10-week old, male WT *SMTNL1*^+/+^ and KO *SMTNL1*^−/−^ mice. Arteries were pressurized to 20 mmHg and treated with increasing concentrations of phorbol 12,13-dibutyrate (**A**, PDBu, 2.5 nM − 1 μM). Cumulative data (% constriction relative to the initial vessel diameter, **B**) were fit with sigmoidal concentration-response curves. Results were analyzed by two-way ANOVA and Sidak’s multiple comparisons test, *statistically significant differences between individual WT and KO data points (p < 0.05, n = 3 separate animals).

The contractile responses of mesenteric arteries from SMTNL1 KO mice were compared with strain-matched WT responses. We examined the pressure-independent constrictions of vessels pressurized at 20 mmHg which, therefore, were not subject to myogenic tone development. Although there was no difference in initial vessel diameters when pressurized to 20 mmHg (WT, 120.2 ± 10.1 μm *vs*. KO, 118.0 ± 5.3 μm), the general concentration-response effects on vessel constriction were modified with SMTNL1 deletion. Vessels from SMTNL1 KO male mice were significantly less responsive to phenylephrine (Fig. 5A), mirroring previous findings obtained with aortic smooth muscle rings^5^. At concentrations above 0.5 μM phenylephrine, the vessel constriction developed by SMTNL1 KO arteries was significantly attenuated relative to that generated in the control arteries (32.8 ± 4.1% *vs*. 18.0 ± 4.1% of initial diameter for WT and KO arteries, respectively). Data were fit with sigmoidal response curves to estimate EC50 values, and SMTNL1 deletion was found to result in only a slight rightward shift in EC50, from 0.95 ± 0.03 µM in the WT to 1.35 ± 0.01 µM in the KO. Concentration-response effects to phenylephrine were also examined in female animals, but no impact of SMTNL1 deletion was observed (Fig. 5B). Subsequently, vessels were subjected to increasing KCl concentration in the bath superfusate (Fig. 6). With deletion of SMTNL1, vessels from male animals were more responsive and developed greater maximal constrictions, reaching 47.2 ± 0.7 in the KO versus only 36.9 ± 2.2 in the WT (% of initial diameter). Finally, the impact of PKC activation with phorbol ester was examined (Fig. 7). Treatment with PDBu elicited significantly greater constrictions of KO vessels when compared to WT, reaching 44.0 ± 5.2% in the KO versus only 33.8 ± 1.6% in the WT. The increase in maximal response of KO vessels was also associated with a reduction in the concentration required for 50% of maximal constriction (EC50 values: WT, 31.5 ± 8.9 μM; KO, 22.8 ± 2.0 μM) suggesting potentiation of PKC signaling in the KO vessels.

## Discussion

Global SMTNL1 deletion in mice was well tolerated, with no overt effects on animal health and no significant alterations in mesenteric artery morphology. However, mesenteric artery contractility in response to KCl-induced depolarization and PDBu-induced activation of PKC was dramatically enhanced in KO male animals with a coincident increase in myogenic tone development. This phenotype was associated with a potent reduction in MYPT1 expression and a significant increase in CPI-17 expression. The combined effect of these two alterations would be to drive a low MLCP:MLCK activity ratio and promote Ca^2+^-sensitization of contractile properties. Taken together, the findings highlight the critical integration of the SMTNL1 protein in microvascular performance. Indeed, it would appear that global deletion of SMTNL1 results in a complex remodeling of signal transduction pathways that govern key contractile processes of the resistance vasculature.

In light of these findings of altered vascular contractility, we completed morphometric examinations of the structure of the KO vessels in comparison to WT controls, as conditions that alter vascular smooth muscle contractility are often associated with vascular remodeling, for instance, inward vessel remodeling in hypertension^24^. An examination of the thoracic aorta using light microscopy and immunohistochemistry revealed no indication of altered vessel diameter or wall thickness, as well as no overt changes in smooth muscle cell hypertrophy or hyperplasia. When third order arteries where mounted and pressurized in Ca^2+^-free buffer, there was no difference in either maximal vessel diameter or wall thickness. Furthermore, when vessels were examined for passive diameters across the full physiological pressure range (10–120 mmHg) no effect of SMTNL1 deletion was observed on internal diameter, wall thickness, distensibility or circumferential stress-strain relationships. These results suggest that any distinctions in contractile nature observed with deletion of SMTNL1 did not arise from structural differences, vascular remodeling, or alterations in vessel wall elasticity. Interestingly, the myogenic responses of the SMTNL1 KO mouse, present on an Sv/129 strain background, appear to more closely resemble those reported for a WT C57Bl/6 animal^16–20^ (Supplemental Fig. S1). Thus, it is important that any comparison of results provided herein for SMTNL1 KO should take into careful consideration and acknowledge the strain differences.

SMTNL1 is known to participate in signaling mechanisms for Ca^2+^ sensitization and desensitization of smooth muscle^1^, and SMTNL1 deletion was shown to impact upon aortic smooth muscle contractility^5,8^. SMTNL1 deletion was previously found to elicit a significant reduction in the maximal contractile force generated in aortic rings in response to PE, with no change in EC50^5,8^. The data collected herein suggest similar impacts of SMTNL1 deficiency on the pharmacomechanical coupling networks in the resistance vasculature. SMTNL1 was again associated with sexual dimorphism of physiological responses; its effects on myogenic reactivity were specific to male animals, with no impact of SMTNL1 deletion observed on vessels isolated from female animals. SMTNL1 has been shown to inhibit MLCP *in vitro*^2,5,8^, and in light of accumulating evidence for the role of Ca^2+^ sensitization as well as defined roles for MLCP inhibition in the myogenic response^9,25,26^, we hypothesized that the vascular myogenic response would be significantly impacted by SMTNL1 deletion. Indeed, third-order mesenteric arteries from male SMTNL1 KO vessels exhibited significant enhancement of the myogenic response, by about 10–20%, over a pressure-range of 60 to 120 mmHg. Moreover, it is likely that the effects of SMTNL1 deletion extend throughout the resistance microvasculature since cerebral arteries from KO animals also exhibit some enhancement of myogenic reactivity^10^. One would predict based on available *in vitro* data that the removal of SMTNL1, given its inhibitory properties *in vitro* toward MLCP, should have elicited a reduction in myogenic tone. However, differences in protein expression discussed in subsequent paragraphs would appear sufficient to explain the effects observed herein for the altered myogenic responses of KO vessels.

Altered protein expressions in the mesenteric arteries of SMTNL1 KO mice suggest that the balance between MLCK and MLCP would be shifted considerably in favour of the kinase and the potential for LC20 phosphorylation and vessel constriction. Of particular significance were observations of increased CPI-17 protein and MYPT1 deficiency in myogenic resistance vessels of the SMTNL1 KO. Changes in the abundance of MYPT1 and CPI-17 proteins are often associated with alterations in vascular contractile properties, including but not limited to: an increase in MYPT1 expression in the pulmonary vasculature with pulmonary arterial hypertension in the rat^27,28^, a decrease in MYPT1 and CPI-17 in mesenteric arteries from a mouse model of sepsis^29^, a decrease in aortic CPI-17 protein in the spontaneously hypertensive rat^30^, and a decrease in MYPT1 in mesenteric vessels following acute myocardial ischemia in rats^31^. Interestingly, an increase in MYPT1 protein was observed in thoracic aorta with SMTNL1 KO^8^. This contrasts with the relationship observed herein for SMTNL1 and MYPT1 in various resistance vessels (Fig. 2C); whereas, an inverse relationship of MYPT1 with SMTNL1 was also similarly observed in larger capacitance vessels, such as the abdominal aorta and the femoral artery (Supplemental Fig. S5). Previously, CPI-17 transcription was found to be suppressed in response to the proliferative stimulus, whereas it was elevated in response to inflammatory, stress-induced stimuli (e.g., transforming growth factor (TGF)-β, interleukin (IL)−1β, tumor necrosis factor (TNF)-α)^32^. These effects on CPI-17 expression are intriguing given that SMTNL1 has been implicated in additional processes that are integral to microvascular function, including permeability, inflammatory and pro-resolving immune responses and extravasation of circulating leukocytes (MacDonald lab, unpublished data). While it remains unresolved why distinct MLCP signaling responses are driven by SMTNL1, the results of the current investigation are reflective of a more complex integration of SMTNL1 and MLCP signals, with regulatory adaptation that is likely dependent upon the vessel bed and perhaps any physiological stressors.

The two dominant regulatory mechanisms for the acute modulation of MLCP activity are MYPT1 phosphorylation (providing either activation or inhibition of the phosphatase depending upon the sites phosphorylated; reviewed in^33,34^) and the phosphorylation of CPI-17 (which becomes a very potent inhibitor of MLCP^23^). It was surprising that the lack of detectable MYPT1 protein in various resistance arteries of the SMTNL1 KO mouse was not associated with more consequential phenotypic changes, given that a lack of MYPT1 and uncoupled MLCP activity could be predicted to allow for maximal LC20 phosphorylation and irreversible vessel constriction. Studies of MYPT1 gene knockout in mice support its role in the regulation of MLCP activity; however, smooth muscle-specific knockout of MYPT1 in the mouse is only associated with modest phenotypic changes to normal vascular function. In this regard, Qiao and colleagues recently demonstrated that MYPT1 deletion had no deleterious effect on cardiac and renal functions or vascular structure, but its deficiency did appear sufficient to elevate systolic blood pressure by ~20 mmHg^35^. In contrast, SMTNL1 deficiency was not associated with any alteration in blood pressure^5^, so additional compensatory mechanisms to maintain vascular homeostasis may be activated in this animal model. The genetic deletion of MYPT1 resulted in the development of increased contractile force by mesenteric arteries in response to membrane depolarization and several contractile agonists^35^. Likewise, SMTNL1 knockout and the coincident suppression of MYPT1 expression were associated with increased mesenteric vessel constrictions in response to KCl-induced membrane depolarization. PKC and ROCK pathways continue to serve as important regulators of contractile processes in both MYPT1- and SMTNL1-deficient animals. However, the SMTNL1 KO vessels did not completely mirror the signaling characteristics of the vascular-targeted smooth muscle MYPT1 knockout. In the MYPT1 knockout mice, the inhibition of ROCK and PKC were shown to equally block agonist-induced contractions of vascular smooth muscle^35^. In the SMTNL1 KO mice, ROCK provided important regulatory control of myogenic constrictions (albeit at luminal pressures >80 mmHg) whereas PKC signaling displays augmented contributions across the 60–120 mmHg pressure range. Taken together, our results are consistent with reports that contractile responses of small mesenteric resistance arteries are mediated primarily by PKC-dependent phosphorylation of CPI-17^36,37^ rather than inhibitory MYPT1 phosphorylation^35^.

The identification of enhanced CPI-17 expression in the SMTNL1 KO mesenteric arteries led to further investigation of PKC signaling in the myogenic response. PKC inhibition has been previously shown to attenuate myogenic reactivity^26,38^. The impact of PKC inhibition on the mesenteric myogenic response was evaluated with GF109203x; while no effect on WT vessels was identified, a significant ~20% attenuation of myogenic reactivity was found for the KO vessels. The effect of pharmacological activation of PKC signaling was also examined, and the contractile response to PDBu was significantly enhanced in KO vessels. CPI-17 phosphorylation was not enhanced by pressure-induced myogenic constriction of rat cerebral arteries^39^ or cremaster arteries^26^. Taken together, these results suggest that the contribution of PKC to myogenic reactivity is enhanced with silencing of SMTNL1. Whether it is through phosphorylation of CPI-17 remains to be determined. Furthermore, any physiological adaptation that impacts upon SMTNL1 expression (e.g., endurance exercise^5^, pregnancy^8^, or development/aging^8^), as well as sexual dimorphic expression^5^, would be predicted to result in a shift in contractile signaling from ROCK to PKC. Since SMTNL1 has the potential to impact upon the expression of genes under the regulation of the progesterone receptor^6^, loss of the transcriptional program associated with SMTNL1 may also have unforeseen impacts on the myogenic response of different vascular beds.

There is emerging recognition that the sexual identity of cells, tissues, and animal model systems is a potent variable within experimental studies^40^. This necessitates that many findings regarding smooth muscle biology require more careful evaluation. There are only a few reports in the literature that address the question of whether male and female resistance vessels exhibit unique myogenic characteristics^16,41,42^. Sex was found to have no impact on the myogenic responses of mesenteric arteries in C57Bl/6 mice; however, only male animals displayed a reduction in tone with hypertension elicited by a high salt diet^16^. In other studies, males and ovariectomized females (again in C57Bl/6 mice) displayed augmented myogenic reactivity of middle cerebral^41^ and mesenteric arteries^42^. The observed effects were linked to estrogen and its impact on endothelial-dependent vasodilation. Importantly, these studies have not yet revealed critical distinctions at the intracellular level that may help to explain sex differences in function at an organismal level. Although further investigation is certainly warranted, our findings as well as other published data^5–8^ indicate that sex dimorphism associated with SMTNL1 could provide differential contributions to cardiovascular phenotypes in males and females.

## Electronic supplementary material

Supplementary Figure S1, Supplementary Figure S2, Supplementary Figure S3, Supplementary Figure S4, Supplementary Figure S5, Supplementary Figure S6
