# Supplementary material for: Smoothelin-like 1 deletion enhances myogenic reactivity of mesenteric arteries with alterations in PKC and myosin phosphatase signaling
Source: Sci Rep. 2019 Jan 24;9:481. doi: 10.1038/s41598-018-36564-0 (PMC6346088; doi:10.1038/s41598-018-36564-0)

SUPPLEMENTARY INFORMATION PROVIDED FOR:

Smoothelin-like 1 deletion enhances myogenic reactivity of mesenteric arteries with alterations in PKC and myosin phosphatase signaling.

Sara R. Turner[1], Mona Chappellaz[1], Brittany Popowich[1], Anne A. Wooldridge[2,#], Timothy A.J. Haystead[2], William C. Cole[3], and Justin A. MacDonald[1,*]

Departments of Biochemistry & Molecular Biology[1] and Physiology & Pharmacology[3], Cumming School of Medicine, University of Calgary, Calgary, AB, T2N 4Z6, Canada
[2]Department of Pharmacology & Cancer Biology, Duke University Medical Center, Durham, NC, 27710, USA

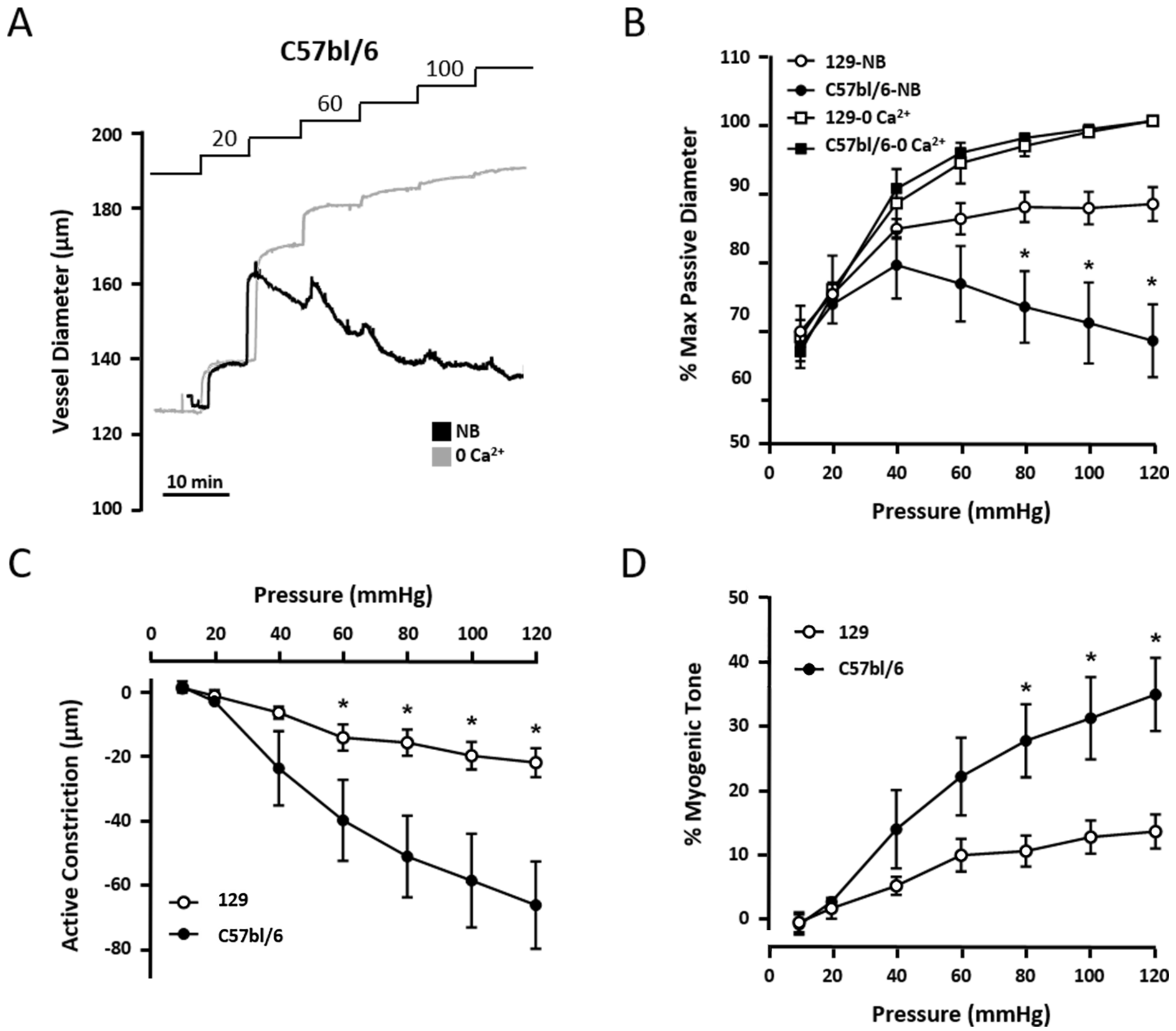

Supplemental Figure S1. Distinct myogenic responses are observed for third-order mesenteric arteries isolated from Sv129 and C57Bl/6 mice. In (A), representative traces show the myogenic responses of third-order mesenteric vessels from adult male C57Bl/6 mice in normal Krebs buffer (NB, black lines) and Ca^{2+}-free Krebs' buffer (0 Ca^{2+}, grey lines). Cumulative data show myogenic responses of vessels from C57Bl/6 (n = 4) and Sv129 (n = 14) mice: % maximum passive diameter (B), the magnitude of active constriction (C), and the % myogenic tone (D). Data were analysed by two-way ANOVA and Sidak's multiple comparisons test. *-statistically significant differences between Sv129 and C57Bl/6 values ($p<0.05$)

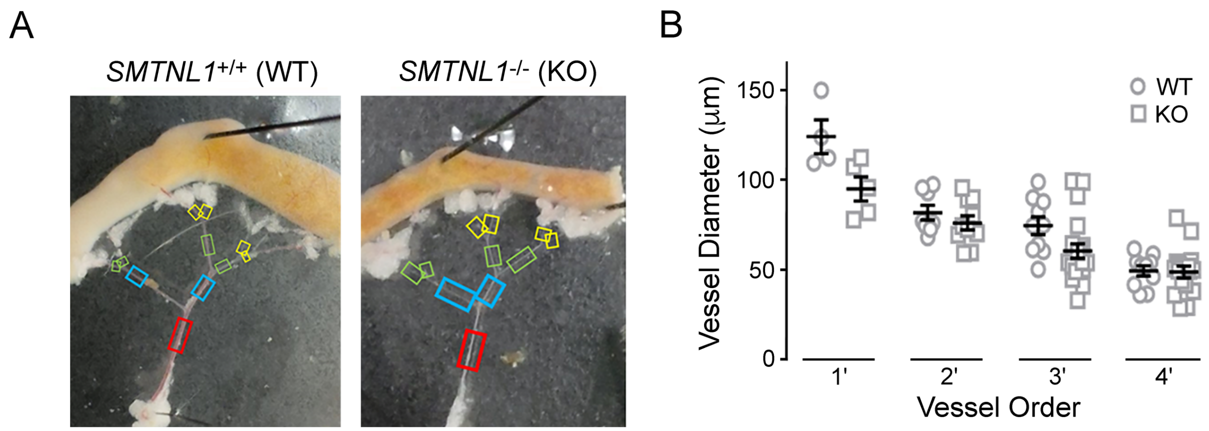

Supplemental Figure S2. Morphometric characteristics of the mesenteric vascular tree. In (A), the arterial branching network of first- (1’, red box), second- (2’, blue box), third- (3’, green box) and fourth-order (4’, yellow box) was visually inspected. Intact mesenteric trees were dissected from the superior mesenteric artery to the ileum. Representative WT and KO trees are shown for 10-week old male animals. In (B), unpressurized vessel diameters were measured as the average over 300 μm of length with an Ion Optix edge detection system. Vascular trees were pinned taught without stretching in Ca^{2+}-free Krebs’ solution.

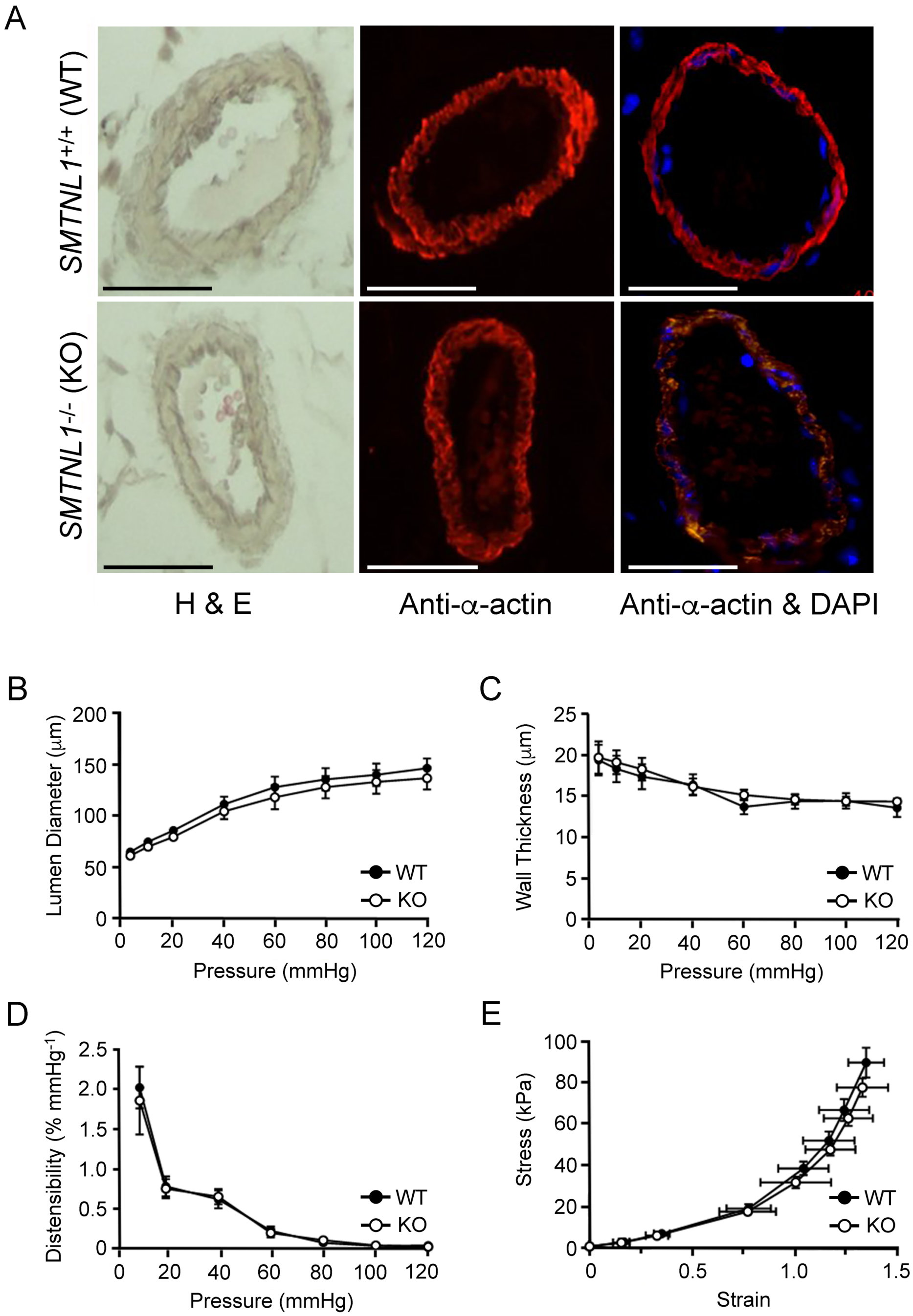

Supplemental Figure S3. SMTNL1 deletion has no effect on mesenteric artery morphology or wall dynamics. Third-order mesenteric arteries were isolated from male WT (n = 4) and KO (n = 5) mice. Histological examination of third-order mesenteric vessels was completed following H&E staining and immunostaining of α-smooth muscle actin (A). Scale bar = 50 µm. Vessels were pressurized in Ca2+-free normal Krebs' buffer in order to measure inner wall diameter (B), vessel wall thickness (C, outer diameter-inner diameter), vessel incremental distensibility (D) and the circumferential stress/strain relationship (E). Data were analyzed by two-way ANOVA, and no significance was reported between WT and KO groups ($p > 0.05$).

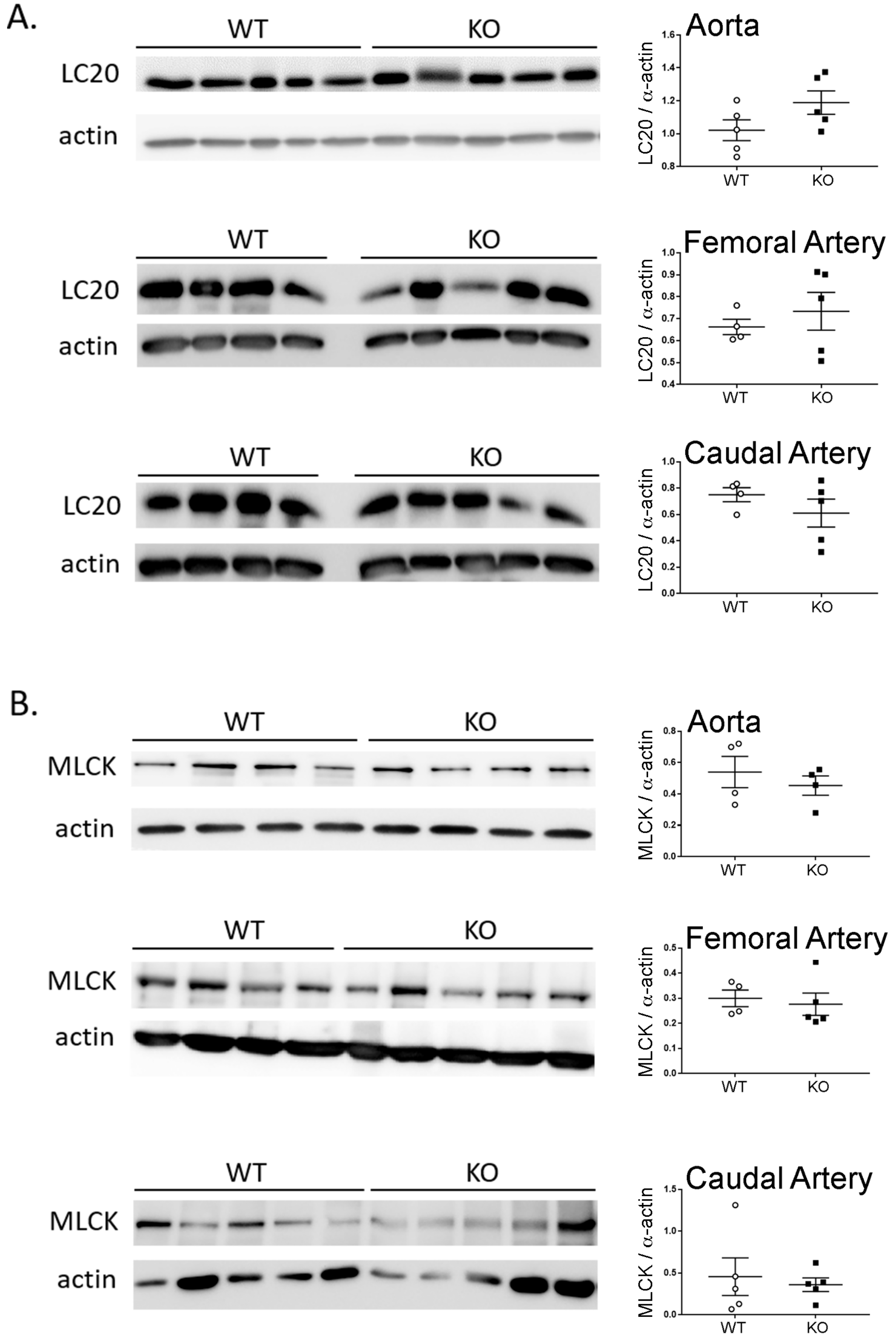

Supplemental Figure S4. Effect of SMTNL1 deletion on LC20 and MLCK levels in the mouse vasculature. LC20 (A) and MLCK (B) protein levels were examined in tissue extracts prepared from abdominal aorta, femoral artery and caudal artery. Tissues were dissected from 10-week old, male animals.

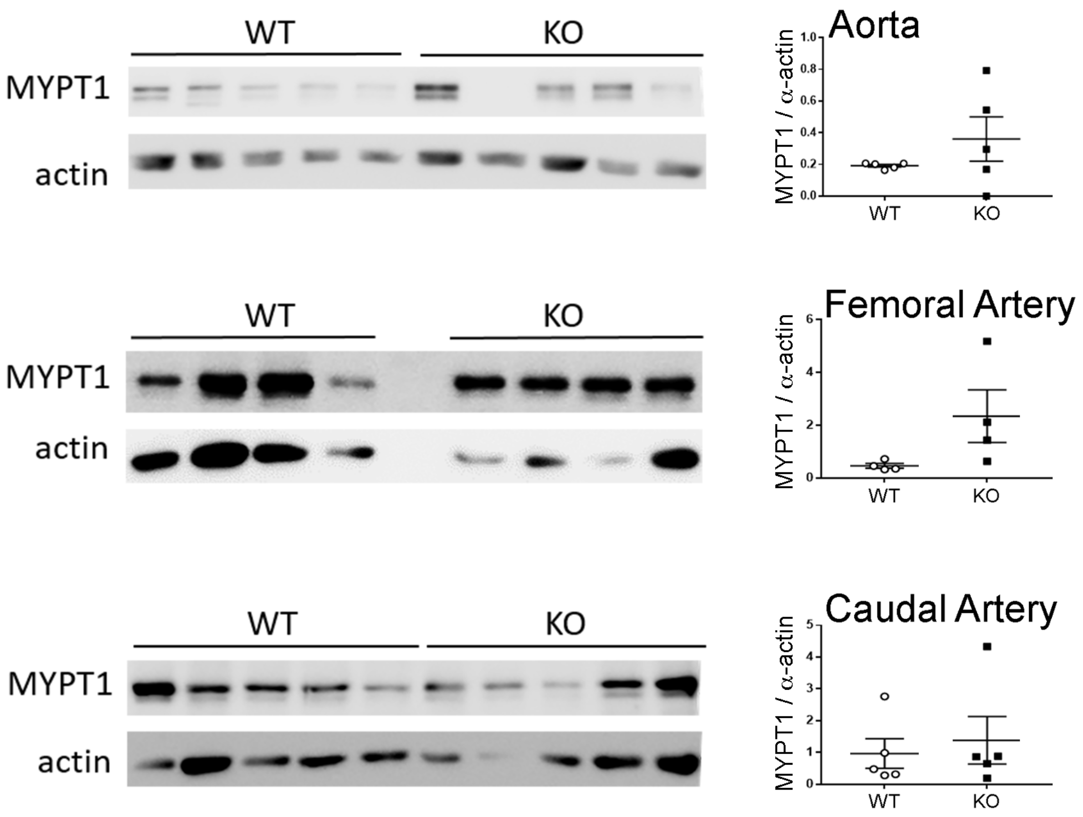

Supplemental Figure S5. Effect of SMTNL1 deletion on MYPT1 levels in the mouse vasculature. MYPT1 protein levels were examined in tissue extracts prepared from abdominal aorta, femoral artery and caudal artery. Tissues were dissected from 10-week old, male animals.

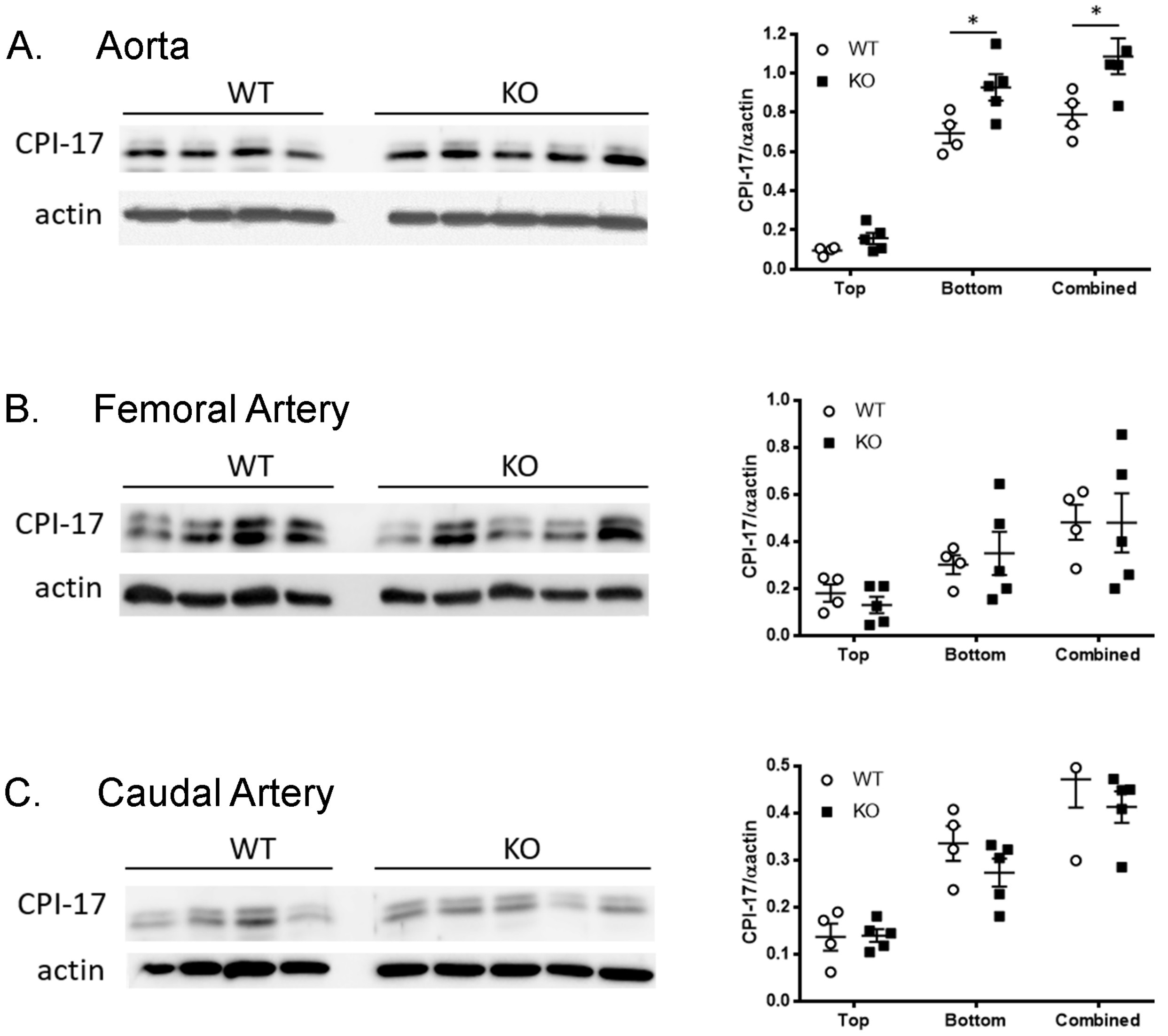

Supplemental Figure S6. Effect of SMTNL1 deletion on CPI-17 levels in the mouse vasculature. CPI-17 protein levels were examined in tissue extracts prepared from abdominal aorta (**A**), femoral artery (**B**) and caudal artery (**C**). Tissues were dissected from 10-week old, male animals. In mouse, CPI-17 expression is characterized by the presence of two isoforms. Densitometry was performed on both upper (top) and lower (bottom) bands. In addition, the intensities were summated and provided as the combined signal. Data were evaluated using the Student's t-test. *- indicates statistically significant differences between WT and KO, $p < 0.05$. Independent tissue samples were dissected from WT ($n = 4$) and KO ($n = 5$) animals.

Supplemental Materials. Full-size western blot images of LC20, MYPT1, CPI-17, MLCK and α-actin protein levels for third-order mesenteric arteries as provided in Figure 2. Samples of rat tail artery (RTA) were used as positive loading controls. Membranes were cut into thirds so that α-actin, LC20 and MYPT1 could be independently probed with primary antibody. MLCK was probed on a full-size membrane. CPI-17 and α-actin were probed on separate membranes since the SDS-PAGE and transfer conditions were distinct for each.

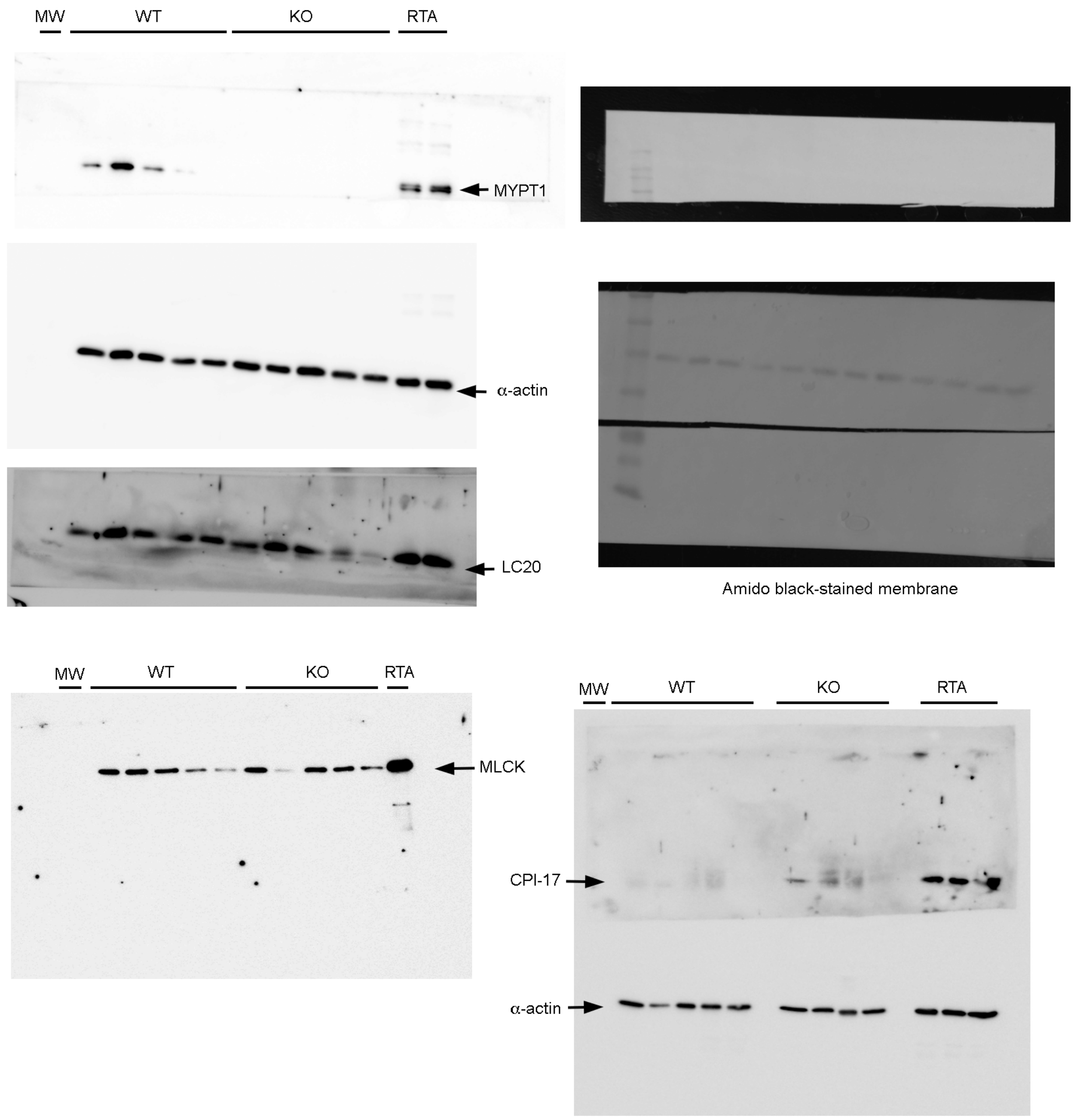

Supplemental Materials. Full-size western blot images of MYPT1 and α-actin protein levels for middle and posterior cerebral arteries (MCA and PCA) as provided in Figure 2. Samples of rat tail artery (RTA) were used as positive loading controls. Membranes were cut in half so that α-actin and MYPT1 could be independently probed with primary antibody.

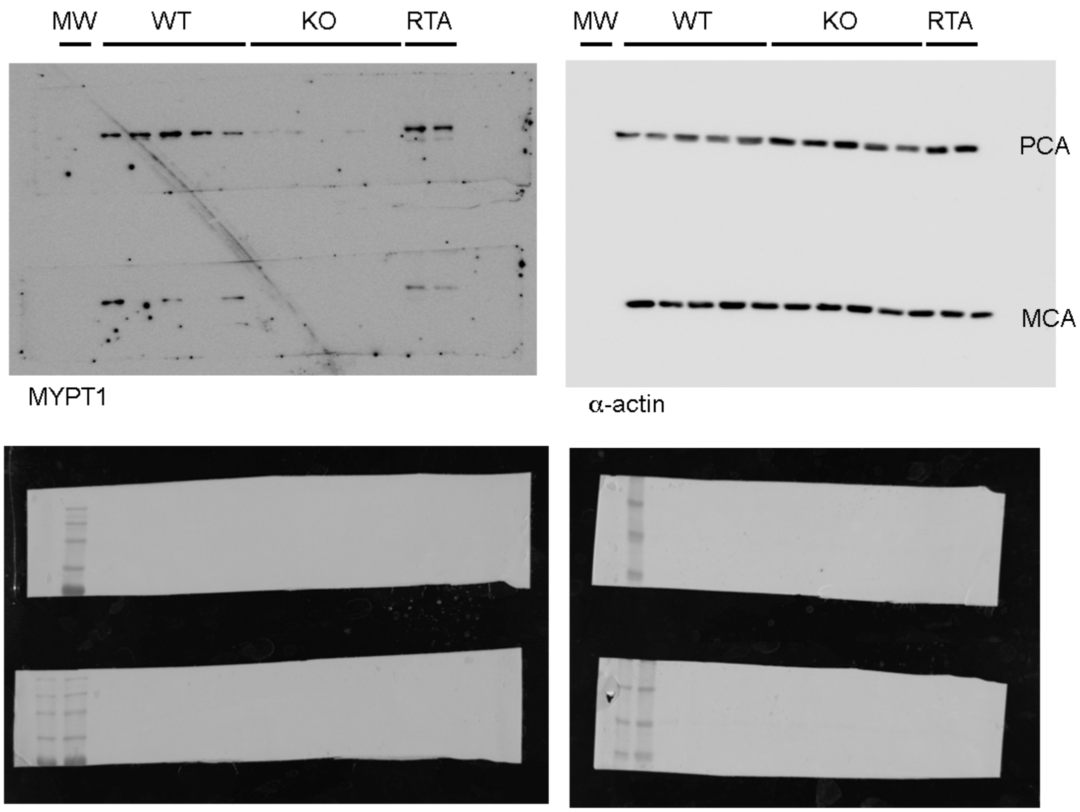

Amido black-stained membranes

Supplemental Materials. Full-size western blot images of LC20 and MLCK protein levels as provided in Supplemental Figure S4. Samples of rat tail artery (RTA) were used as positive loading controls. Membranes were cut in half so that α-actin and MLCK or LC20 could be independently probed with primary antibody.

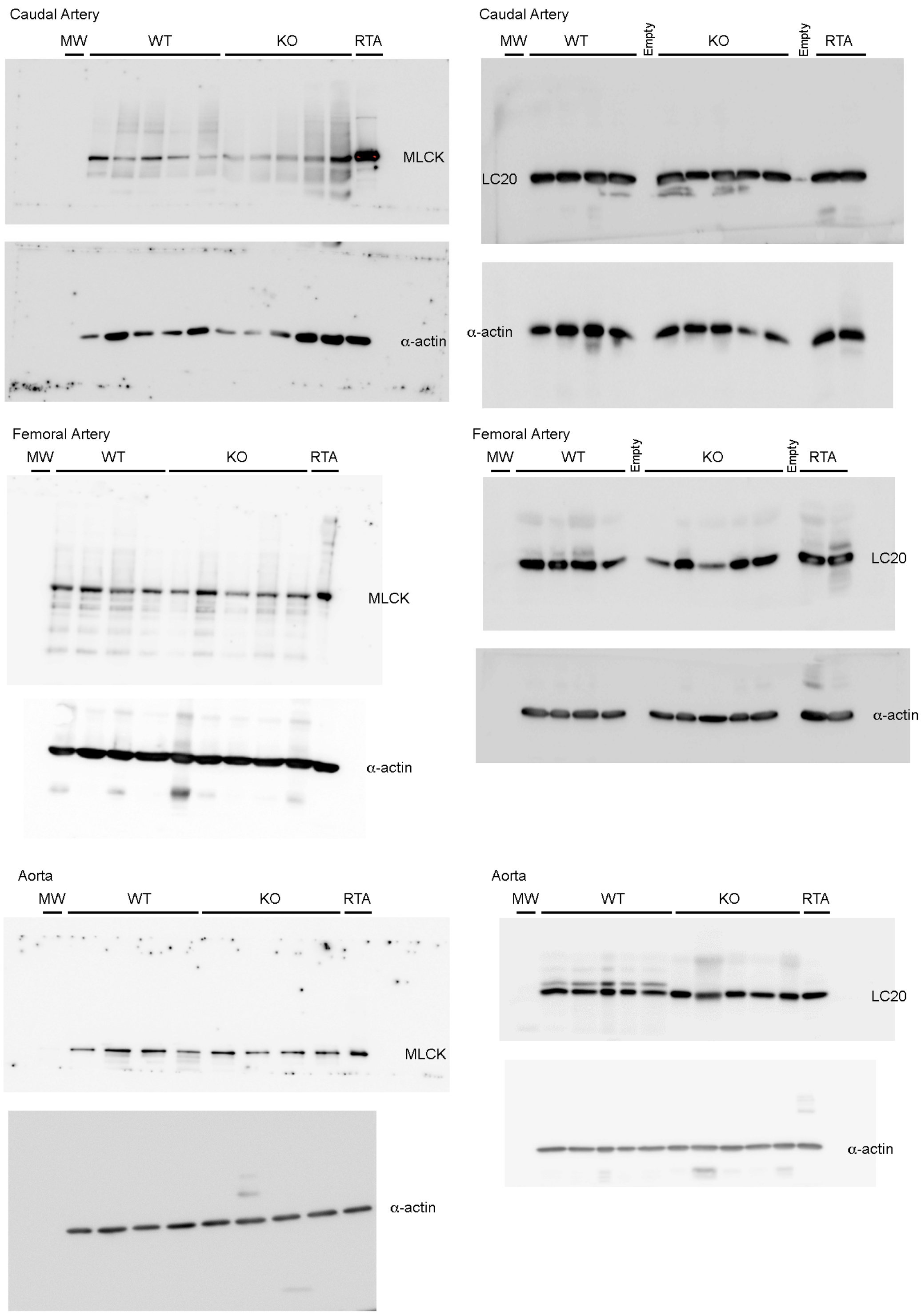

Supplemental Materials. Full-size western blot images of MYPT1 protein levels as provided in Supplemental Figure S5. Samples of rat tail artery (RTA) were used as positive loading controls. Membranes were cut in half so that MYPT1 and α-actin could be independently probed with primary antibody.

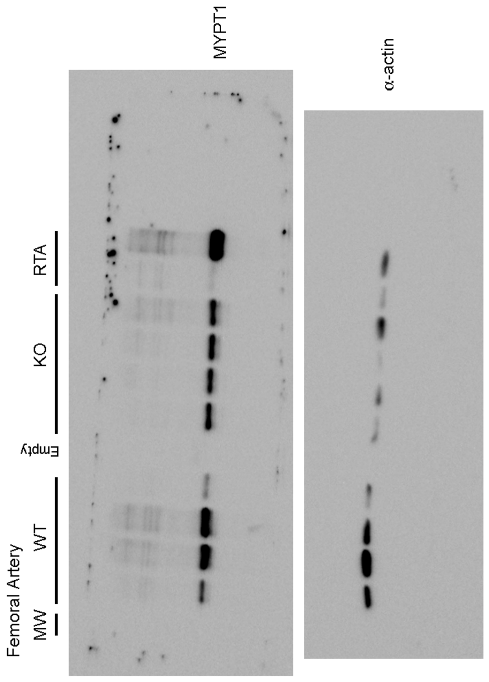

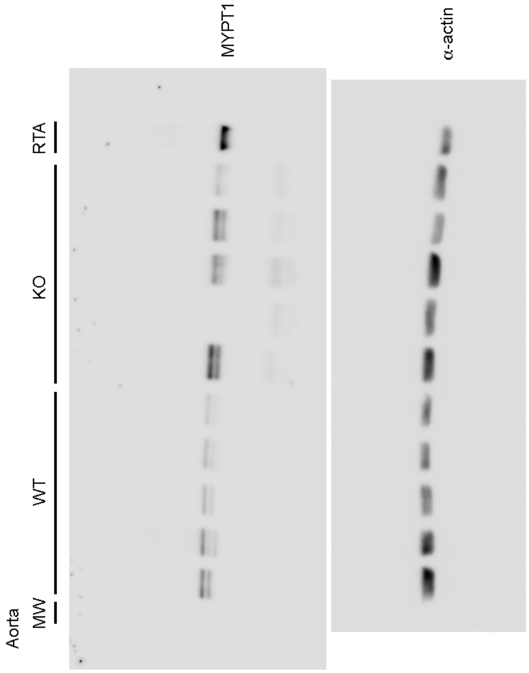

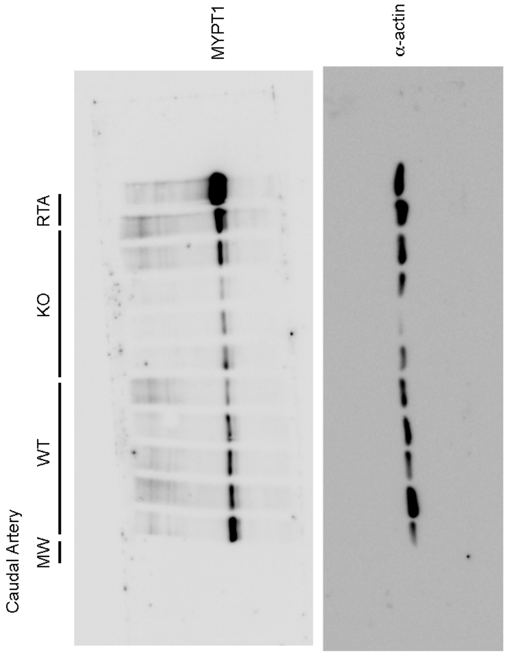

Supplemental Materials. Full-size western blot images of CPI-17 protein levels as provided in Supplemental Figure S6. Samples of rat tail artery (RTA) were used as positive loading controls. Membranes were cut in half so that CPI-17 and α-actin could be independently probed with primary antibody.

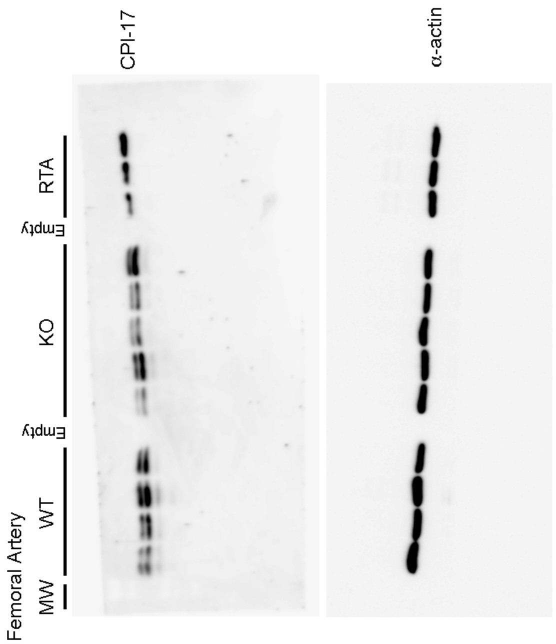

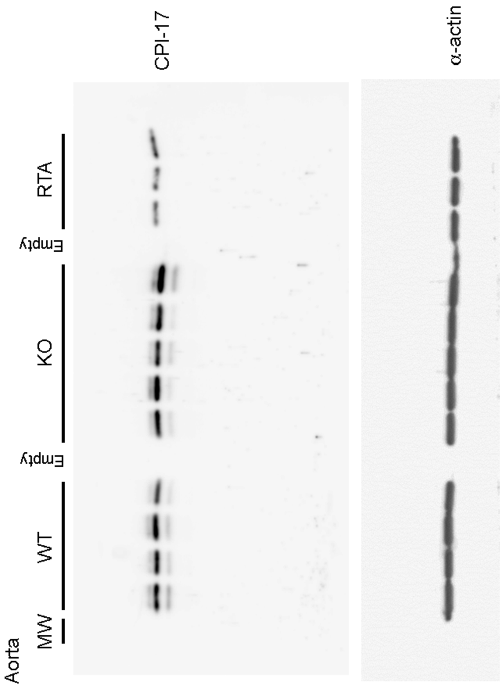

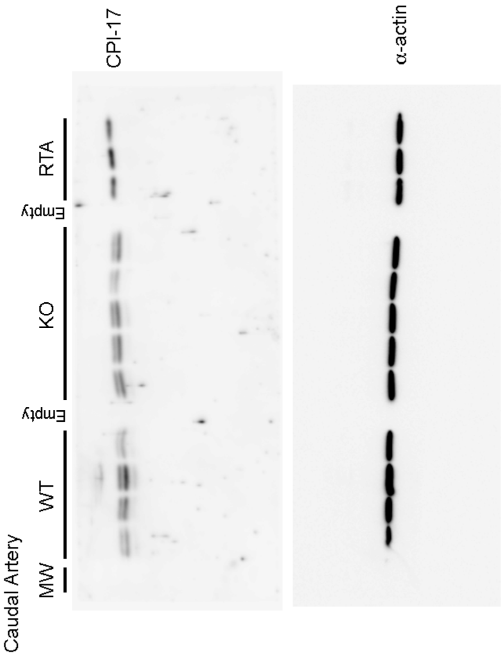

Supplement: Supplementary file 1 — Supplementary Figure S1, Supplementary Figure S2, Supplementary Figure S3, Supplementary Figure S4, Supplementary Figure S5, Supplementary Figure S6 [file 41598_2018_36564_MOESM1_ESM.pdf]
